# Pharmacology, safety, efficacy and clinical uses of the COX‐2 inhibitor robenacoxib

**DOI:** 10.1111/jvp.13052

**Published:** 2022-04-22

**Authors:** Peter Lees, Pierre‐Louis Toutain, Jonathan Elliott, Jerome M. Giraudel, Ludovic Pelligand, Jonathan N. King

**Affiliations:** ^1^ Royal Veterinary College University of London London UK; ^2^ INTHERES, INRA, ENVT Université de Toulouse Toulouse France; ^3^ Le Bouscat France; ^4^ J.N.King Consultancy Bennwil Switzerland

**Keywords:** cat, coxib, dog, NSAID, robenacoxib

## Abstract

Robenacoxib is a veterinary‐approved non‐steroidal anti‐inflammatory drug (NSAID) of the coxib group. It possesses anti‐hyperalgesic, anti‐inflammatory and anti‐pyretic properties. Robenacoxib inhibits the cyclooxygenase (COX)‐2 isoform of COX selectively (in vitro IC_50_ ratios COX‐1:COX‐2, 129:1 in dogs, 32:1 in cats). At registered dosages (2 mg/kg subcutaneously in dogs and cats, 1–4 mg/kg orally in dogs and 1–2.4 mg/kg orally in cats), robenacoxib produces significant inhibition of COX‐2 whilst sparing COX‐1. The pharmacokinetic (PK) profile of robenacoxib is characterized by a high degree of binding to plasma proteins (>98%) and moderate volume of distribution (at steady state, 240 ml/kg in dogs and 190 ml/kg in cats). In consequence, the terminal half‐life in blood (<2 h) is short, despite moderate body clearance (0.81 L/kg/h) in dogs and low clearance (0.44 L/kg/h) in cats. Excretion is principally in the bile (65% in dogs and 72% in cats). Robenacoxib concentrates in inflamed tissues, and clinical efficacy is achieved with once‐daily dosing, despite the short blood terminal half‐life. In dogs, no relevant breed differences in robenacoxib PK have been detected. Robenacoxib has a wide safety margin; in healthy laboratory animals daily oral doses 20‐fold (dog, 1 month), eight‐fold (cat, 6 weeks) and five‐fold (dog, 6 months) higher than recommended clinical doses were well tolerated. Clinical efficacy and safety have been demonstrated in orthopaedic and soft tissue surgery, and in musculoskeletal disorders in dogs and cats.

## 1 INTRODUCTION AND CHEMISTRY

Robenacoxib is a non‐steroidal anti‐inflammatory drug (NSAID) of the coxib class. The coxibs were introduced with the objective of selectively inhibiting the cyclooxygenase‐2 (COX‐2) isoform of COX. COX‐1 is present in many tissues constitutively and has several protective functions, including gastric cytoprotection, regulation of renal blood flow and regulation of platelet activity, whilst COX‐2 is mainly induced locally and for restricted periods, and is primarily responsible for pain and inflammation (Pairet & Engelhardt, 1996). Drugs that inhibit COX‐2 but spare COX‐1 were therefore designed to have improved safety margins, especially for the gastrointestinal tract (Flower, 2003).

**FIGURE 1:**
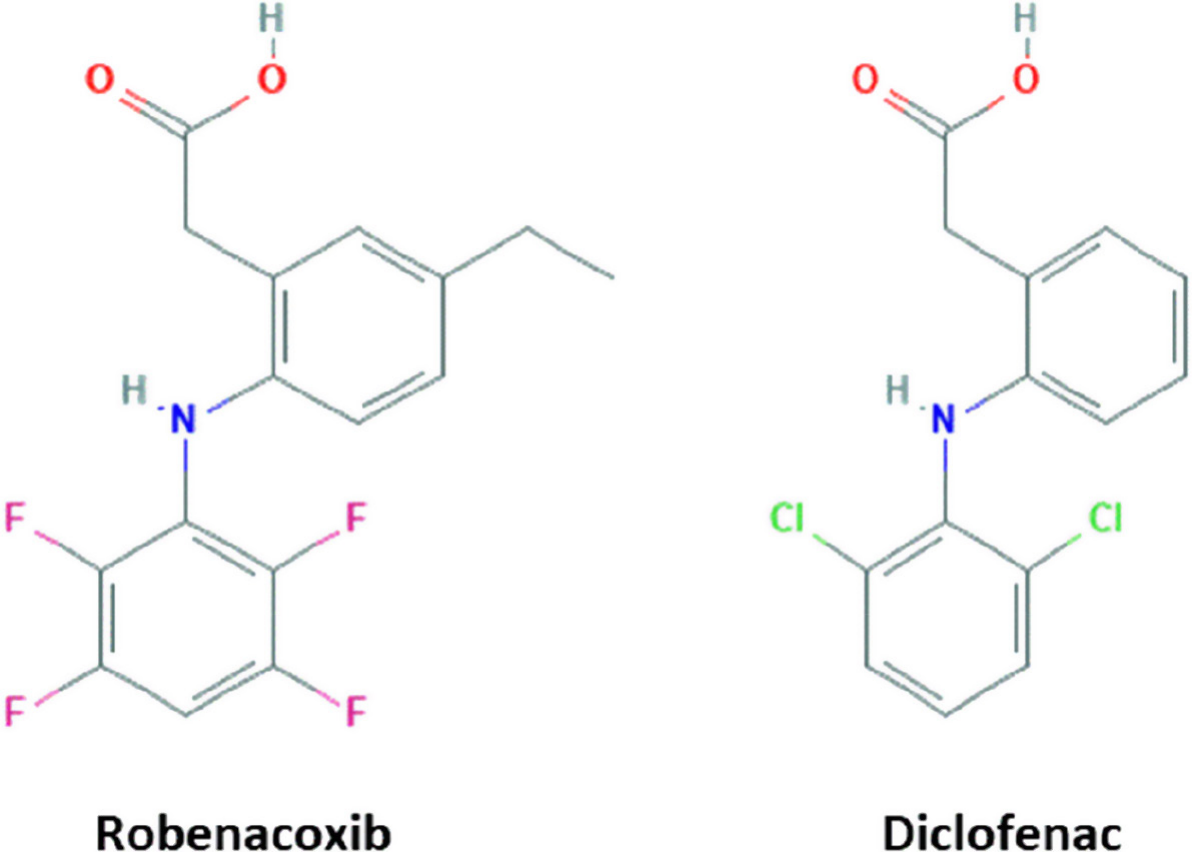
Chemical structures of robenacoxib and diclofenac. Compared with diclofenac, robenacoxib contains an additional ethyl group at the five position on the phenyl ring and four fluorine atoms replace two chlorine atoms

Robenacoxib is a highly selective COX‐2 inhibitor and is registered as injectable and flavoured tablet formulations for dogs and cats (Table 1). Robenacoxib has the IUPAC name 2‐[5‐ethyl‐2‐(2,3,5,6‐tetrafluoro‐phenylamino)‐phenyl]‐acetic acid; chemical formula C_16_H_13_F_4_NO_2_; and molecular mass 327.279 g/mol. It is chemically related to diclofenac, an older NSAID with moderate selectivity for COX‐2 (Figure 1) (King et al., 2009). Unlike most selective COX‐2 inhibitors, robenacoxib contains a carboxyl group and lacks a sulphur‐containing group. It therefore differs chemically, and has different pharmacological properties, from the sulphone firocoxib and the sulphonamide‐containing (cimicoxib, deracoxib, enflicoxib, mavacoxib and vitacoxib) coxibs.

**TABLE 1:** Registered indications for robenacoxib in the EU and the United States

EU (www.ema.europa.eu/en/medicines/veterinary/EPAR/onsior, accessed 3 Nov 2021)
Cats (tablets)
For the treatment/relief of pain and inflammation associated with acute and/or chronic musculoskeletal disorders
For the reduction in moderate pain and inflammation associated with orthopaedic surgery
Dogs (tablets)
For the treatment of pain and inflammation associated with chronic osteoarthritis. For the treatment of pain and inflammation associated with soft tissue surgery
Cats and dogs (solution for injection)
For the treatment of pain and inflammation associated with orthopaedic or soft tissue surgery
USA (www.fda.gov, accessed 3 Nov 2021)
Cats (solution for injection and tablets)
For the control of post‐operative pain and inflammation associated with orthopaedic surgery, ovariohysterectomy and castration in cats >5.5 lbs (2.5 kg) and >4 months of age; for up to a maximum of 3 days
Dogs (solution for injection and tablets)
For the control of post‐operative pain and inflammation associated with soft tissue surgery in dogs ≥4 months of age; for up to a maximum of 3 days

## 2 PHARMACODYNAMICS

Although other modes of action cannot be excluded, all important pharmacodynamic (PD) properties of robenacoxib have been attributed to COX‐2 inhibition. Increased molecular bulk and altered shape account for robenacoxib's COX‐2 selectivity (Figures 1, 2).

**IGURE 2:**
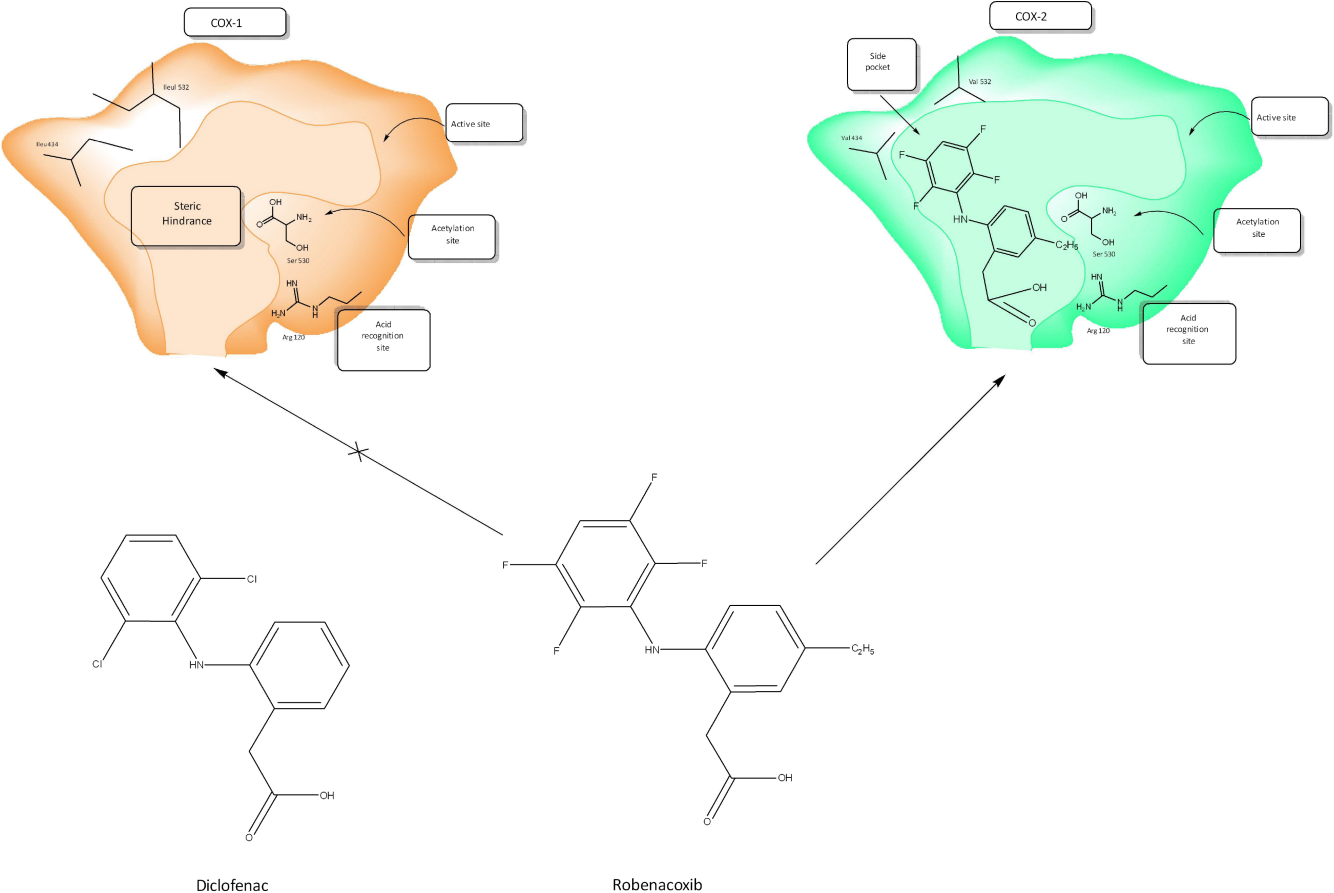
Diagram illustrating presumed selective access of robenacoxib to the COX‐2 but not COX‐1 binding site. In contrast, the less selective analog diclofenac can gain access to both COX‐1 and COX‐2 binding sites. It is likely that robenacoxib resembles lumiracoxib in forming, through the carboxylate group, hydrogen bonds with the catalytic Tyr‐385 and with Ser‐530 on COX‐2, rather than with the larger hydrophobic side pocket (as used by other selective COX‐2 inhibitors) or with Arg‐120 (as used by carboxylate containing non‐selective NSAIDs of the profen subgroup) (Rordorf et al., 2005)

### 2.1 Inhibition of cyclooxygenase

In all species tested, robenacoxib is a potent and selective COX‐2 inhibitor, producing no significant COX‐1 inhibition at clinically recommended dosages.

#### 2.1.1 Non‐target species

In early studies, robenacoxib was evaluated in purified enzyme assays. Binding to ovine COX‐1 was weak and rapidly reversible (dissociation *T*_1/2_ <1 min), whilst binding to human recombinant COX‐2 was potent and slowly reversible (*T*_1/2_ 25 min) (King et al., 2009). Binding affinities were 0.8 µM (COX‐1) and 0.03 µM (COX‐2), indicating both selectivity and high potency for COX‐2 inhibition. Compared with naproxen (non‐selective) and diclofenac (moderately COX‐2 selective), robenacoxib was also highly COX‐2 selective in cell‐based assays (King et al., 2009) (Table 2).

**TABLE 2:** NSAID potency (IC_50_) for inhibition of COX‐1 and COX‐2 in cell‐based assays^a^

Drug	COX‐1 inhibition (IC_50,_ µM)	COX‐2 inhibition (IC_50,_ µM)
Robenacoxib	> 30 (11)	0.031 ± 0.010 (11)
Celecoxib	> 30 (8)	0.80 ± 2.30 (8)
Diclofenac	0.19 ± 0.05 (22)	0.013 ± 0.001 (22)
Naproxen	2.7 ± 4.5 (8)	1.60 ± 1.40 (8)

^a^ Mean ± SD (number of samples) (King et al., 2009). COX‐1 inhibition was determined using stable transfected HEK293 cells. COX‐2 inhibition was determined using IL‐lβ‐stimulated dermal fibroblasts.

Further data were obtained from rats in inflammatory exudate and whole‐blood assays. In the lipopolysaccharide (LPS)‐stimulated air pouch, ID_50_ values for inhibition of COX‐2‐derived prostaglandin (PG)E_2_ were 0.3 mg/kg [robenacoxib, orally (PO)] and 0.1 mg/kg (diclofenac, PO) (King et al., 2009). In the zymosan‐stimulated tissue chamber inflammation model, 2 mg/kg robenacoxib PO inhibited COX‐2 by 83% at 12 h and did not inhibit COX‐1 (King et al., 2009).

In a gastric tolerability study in rats, diclofenac (30 mg/kg PO) inhibited serum thromboxane (Tx)B_2_ and PGE_2_ and 6‐keto‐PGF_1α_ in gastric and ileal biopsies, consistent with COX‐1 inhibition, whereas the same high robenacoxib dosage (30 mg/kg PO) produced no significant changes compared with vehicle (Table 3) (King et al., 2009).

**TABLE 3:** Concentrations of serum TxB_2_ and gastrointestinal PGs in rats after oral dosing of vehicle, robenacoxib and diclofenac

Drug (dosage)	Serum TxB_2_ ^a^	Gastric 6‐keto PGF_1α_ ^b^	Ileal PGF_1α_ ^b^	Gastric PGE_2_ ^b^	Ileal PGE_2_ ^b^
Vehicle (control)	310 ± 32.6	376 ± 275	223 ± 178	58.7 ± 63.3	91.5 ± 53.5
Robenacoxib (30 mg/kg)	180 ± 4.4^§^	283 ± 241^§^	252 ± 153^§§§^	62.7 ± 81.7	96.4 ± 52.3^§§^
Diclofenac (30 mg/kg)	11.2 ± 27*	102 ± 88**	49.3 ± 51.3**	9.7 ± 13.0**	36.1 ± 30.6**

Note

Data are mean ± SD (King et al., 2009).

The significance of differences from vehicle control is indicated by asterisks: *p < .05; **p < .01. The significance of differences between robenacoxib and diclofenac is indicated by ^§^ *p* < .05; ^§§^ *p* < .01; and ^§§§^ *p* < .001.

^a^ ng/ml.

^b^ ng/g wet weight/10 min.

#### 2.1.2 Dog

Whole‐blood COX‐1 and COX‐2 assays are the most relevant to clinical use (Pairet & Engelhardt, 1996; Patrignani et al., 1994). In comparative in vitro whole‐blood assays, IC_50_ values for COX‐1 and COX‐2 indicated non‐selectivity for ketoprofen, moderate COX‐2 selectivity for R‐carprofen, meloxicam, diclofenac and S‐carprofen, and high selectivity for robenacoxib (Table 4) (King et al., 2010). However, IC_50_ values have limited clinical relevance, because concentration inhibition curve slopes for both COX‐1 and COX‐2 are relatively shallow for many NSAIDs (Kay‐Mugford et al., 2000; Lees et al., 2004; Warner et al., 1999). Therefore, even high COX‐1:COX‐2 IC_50_ ratios do not ensure the absence of COX‐1 inhibition at clinically recommended dosages. COX‐2 IC_80_ is more relevant than IC_50_ as a predictor of efficacy, as most NSAIDs inhibit COX‐2 by approximately 80% at clinically effective concentrations (Lees et al., 2004; Warner et al., 1999). This principle is very likely valid also for robenacoxib; in dogs, the ED_80_ for inhibition of COX‐2 (1.21 mg/kg) was virtually identical to the ED_50_ for improvement in weight‐bearing in the urate synovitis model (1.23 mg/kg) (Schmid et al., 2010b). It is advisable to limit COX‐1 inhibition to less than 20%. Therefore, other ratio variants have been determined; robenacoxib was COX‐2‐selective as indicated by the ratio IC_20_ COX‐1:IC_80_ COX‐2 (Table 4) (King et al., 2010).

**TABLE 4:** ICx values for COX‐1 and COX‐2 in canine whole‐blood in vitro assays

Drug	COX‐1 inhibition IC_50_ (µM)	COX‐2 inhibition IC_50_ (µM)	Selectivity for inhibition of COX‐2/COX‐1	
			Quotient IC_50_ COX‐1:IC_50_ COX‐2	Quotient IC_20_ COX‐1:IC_80_ COX‐2
Robenacoxib	10.8	0.079	128.8	19.8
Deracoxib	9.99	0.203	48.5	5.33
Celecoxib	9.22	0.450	20.4	2.34
S(+)‐Carprofen	25.7	1.47	17.6	2.45
R(‐)‐Carprofen	266.6	45.6	5.85	2.07
Diclofenac	0.33	0.030	10.9	1.38
Meloxicam	1.22	0.142	7.42	0.460
Ketoprofen	0.105	0.123	0.881	0.208

Note

Data are geometric means (*n* = 9 or 10) (King et al., 2010). COX‐1 was assessed from TxB_2_ concentration in blood allowed to clot at 37°C, measured by enzyme immunoassay. COX‐2 was assessed from PGE_2_ synthesis in blood samples, incubated in the presence of LPS, measured by enzyme immunoassay.

**FIGURE 3:**
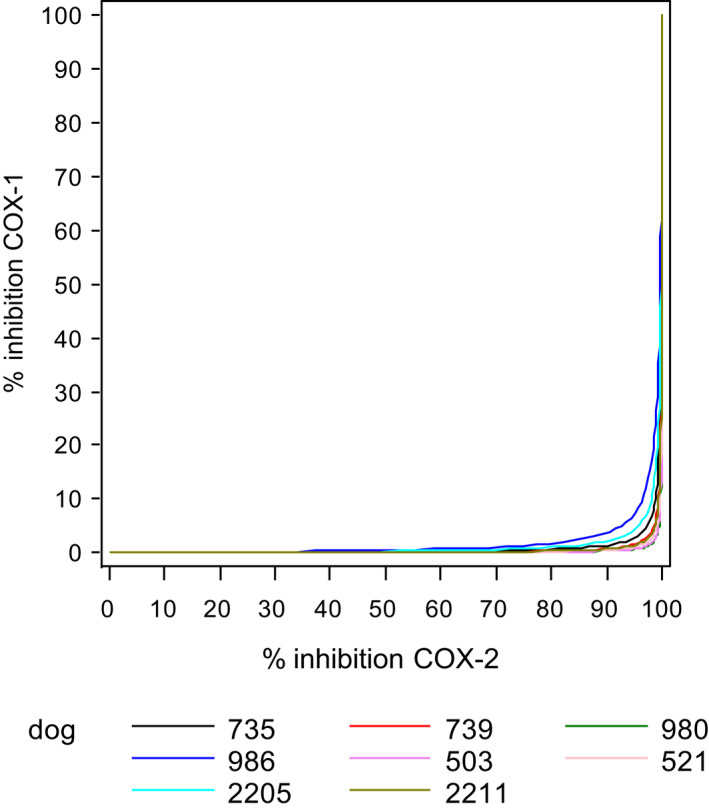
Simulated plots of percentage inhibition of COX‐1 (TxB_2_) versus percentage inhibition of COX‐2 (PGE_2_) for robenacoxib in individual dogs. In vitro study in whole blood (King et al., 2011). TxB_2_ was generated in serum by clotting of whole blood. PGE_2_ was generated in plasma by incubation of blood samples with LPS

Concentrations of robenacoxib inhibiting COX‐2 by 90% produced minimal (<10%) inhibition of COX‐1 (King et al., 2010) (Figure 3).

**FIGURE 4:**
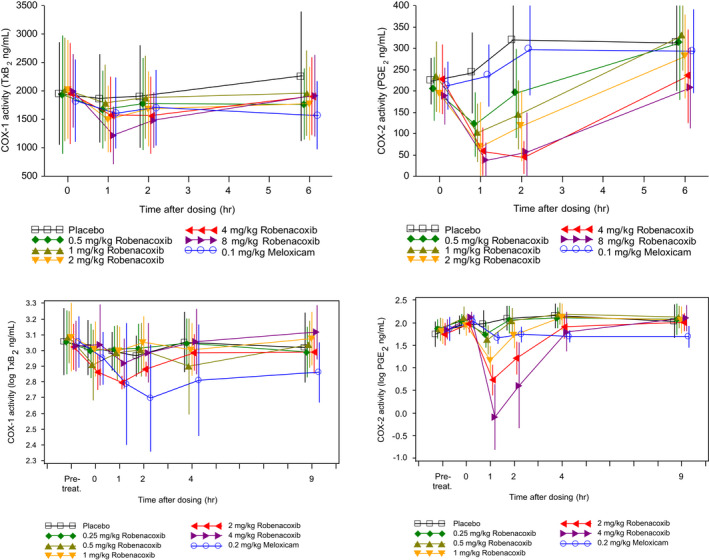
Inhibition in blood in dogs of: COX‐1 activity assessed by serum TxB_2_ generated ex vivo by clotting of whole blood (left); and COX‐2 activity assessed from plasma PGE_2_ concentrations generated ex vivo by incubation of blood samples with LPS (right) (Borer et al., 2017; Schmid et al., 2010b). Single doses of robenacoxib, meloxicam or placebo were administered PO (upper, *n* = 6–8 per group) or SC (lower, *n* = 12 per group). Data are mean ± SD. To add clarity, symbols are distributed around the true time points

**FIGURE 5:**
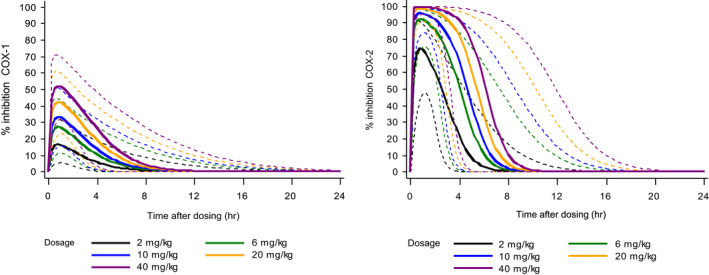
Simulated percentage inhibition of COX‐1 (serum TxB_2_, left) and COX‐2 (plasma PGE_2_, right) in dogs after PO administration of robenacoxib at five dosages (2–40 mg/kg). TxB_2_ was generated in serum ex vivo by clotting of whole blood. PGE_2_ was generated in plasma ex vivo by incubation of blood samples with LPS. Full line = median, dotted lines = 90% tolerance interval (King et al., 2011)

Robenacoxib inhibition of COX isoenzymes was further investigated in in vivo and ex vivo studies, as both safety and efficacy of NSAIDs depend not only on potency for inhibition of COX‐1 and COX‐2 but also on concentrations (magnitude and time course) achieved systemically. In Beagle dogs, COX inhibition in blood was studied after robenacoxib administration at therapeutic and higher dosages (PO 1–8 mg/kg, SC 0.5–4 mg/kg) (Borer et al., 2017; King et al., 2011; Schmid et al., 2010b). All dosages inhibited COX‐2 (Figures 4, 5). However, clinically recommended dosages (PO 1–4 mg/kg, SC 2 mg/kg) produced no COX‐1 inhibition, except transiently at Cmax with the 8 mg/kg PO dosage (Figure 4).

#### 2.1.3 Cat

In in vitro whole‐blood assays, robenacoxib was highly COX‐2‐selective; IC_50_ COX‐1:IC_50_ COX‐2 ratio was 502:1 (Giraudel et al., 2009b).

Robenacoxib was also COX‐2‐selective in an in vitro study, although the IC_50_ COX‐1:IC_50_ COX‐2 ratio of 32:1 was lower than in dogs (129:1) (Tables 4, 5) (Schmid et al., 2010a). In comparison, COX‐2 inhibition was preferential for diclofenac and meloxicam, whilst ketoprofen was COX‐1‐selective (Table 5).

**TABLE 5:** ICx values for COX‐1 and COX‐2 in feline whole‐blood in vitro assays

Drug	COX‐1 inhibition IC_50_(µM)	COX‐2 inhibition IC_50_(µM)	Selectivity for inhibition of COX‐2/COX‐1	
			Quotient IC_50_ COX‐1:IC_50_ COX‐2	Quotient IC_20_ COX‐1:IC_80_ COX‐2
Robenacoxib	4.47	0.139	32.2	4.23
Diclofenac	0.294	0.076	3.88	0.481
Meloxicam	1.36	0.509	2.67	0.250
Ketoprofen	0.023	0.472	0.044	0.0052

Note

Data are geometric means (*n* = 8) (Schmid et al., 2010a).

COX‐1 was assessed from TxB_2_ concentration in blood allowed to clot at 37°C, measured by enzyme immunoassay. COX‐2 was assessed from PGE_2_ synthesis in blood samples, incubated in the presence of LPS, measured by enzyme immunoassay.

Other whole‐blood assay selectivity indices, including the IC_20_ COX‐1:IC_80_ COX‐2 ratio, confirm the high COX‐2 selectivity of robenacoxib in cats (Table 5, Giraudel et al., 2009b). Predicted COX‐1 inhibition was low with robenacoxib in two studies; 5.2% and 7.6% at 90% COX‐2 inhibition (Table 6, Figure 5). Even lower COX‐1 inhibition was predicted for robenacoxib in other studies (Table 7). The IC_80_ COX‐2 for robenacoxib in cats correlated with efficacy against pain, inflammation and fever in the kaolin model (Giraudel et al., 2009a).

**TABLE 6:** Percentage inhibition of COX‐1 for varying percentage inhibitions of COX‐2 in cats in two studies

% inhibition of COX‐1 for inhibition of COX‐2 of 50‐95%				
Drug	50%	80%	90%	95%
Robenacoxib^a^	0.56	2.31	5.17	10.5
Robenacoxib^b^	1.60	4.40	7.60	12.4
Diclofenac^b^	15.5	38.5	56.1	71.2
Meloxicam^b^	27.9	48.9	62.0	72.7
Ketoprofen^b^	97.7	99.4	99.8	99.9

Note

Data are geometric means (*n* = 8).

^a^ Computed with the parameters of the Hill equation for COX‐1 and COX‐2 and determined using a non‐linear parametric mixed‐effects model. COX‐1 and COX‐2 activities, in heparinized blood samples, were induced with calcium ionophore A23187 and LPS, respectively. TxB_2_ was the marker for both COX‐1 and COX‐2 activities (Giraudel et al., 2009b).

^b^ PGE_2_ synthesis in blood samples incubated in the presence of LPS. TxB_2_ concentration in blood allowed to clot at 37°C (Schmid et al., 2010a).

**TABLE 7:** Percentage inhibition of COX‐1 induced by robenacoxib for various percentage inhibitions of COX‐2 in cats in three studies

	Giraudel et al. (2009b)^a^	Pelligand et al. (2012)^b^	Pelligand et al. (2014)^b^
Inhibition of COX‐1 for IC_50_ COX‐2	0.56	0.06	2.5
Inhibition of COX‐1 for IC_80_ COX‐2	2.31	0.50	5.6
Inhibition of COX‐1 for IC_90_ COX‐2	5.17	1.32	ND
Inhibition of COX‐1 for IC_95_ COX‐2	10.5	3.21	12.9
Inhibition of COX‐1 for IC_99_ COX‐2	39.2	19.8	28.2

^a^ COX‐1 and COX‐2 activities induced in vitro in heparinized feline blood samples with calcium ionophore and LPS, respectively. Inhibition of TxB_2_ provided a marker of both COX‐1 and COX‐2 activities (Giraudel et al., 2009b).

^b^ Robenacoxib ex vivo selectivity for COX‐1 (blood allowed to clot in glass tubes) and in vivo selectivity for COX‐2 (tissue cage inflammatory exudate) using serum TxB_2_ (COX‐1) and exudate PGE_2_ (COX‐2) as markers (Pelligand et al., 2012; Pelligand et al., 2014).

**FIGURE 6:**
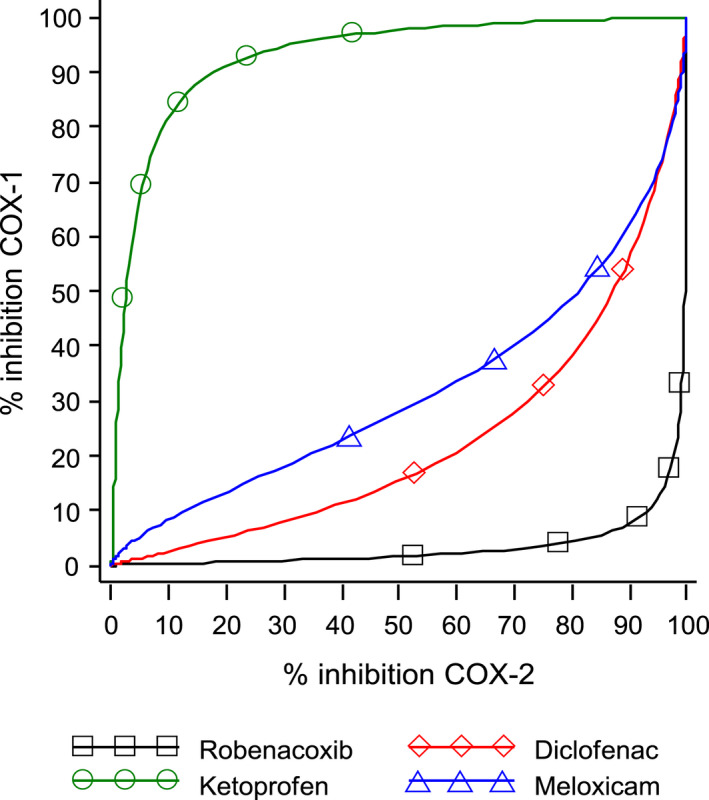
Simulated percentage COX‐1 inhibition (TxB_2_, ordinate) versus percentage COX‐2 inhibition (PGE_2_, abscissa) for four drugs in cats. In vitro study in whole blood (Schmid et al., 2010a). TxB_2_ was generated in serum by clotting of whole blood. PGE_2_ was generated in plasma by incubation of blood samples with LPS

In vivo COX‐2 selectivity of robenacoxib in cats was obtained at clinically recommended dosages (1–2 mg/kg PO, 2 mg/kg SC). COX‐2 inhibition was accompanied by sparing of COX‐1 (Figure 6) (Schmid et al., 2010b).

#### 2.1.4 Horse

Robenacoxib was COX‐2‐selective in the horse, indicated by an IC_50_COX‐1:IC_50_COX‐2 ratio of 61:1 in in vitro whole‐blood assays (Marshall et al., 2011).

Thus, in all species tested (cats, dogs, horses and rats) robenacoxib selectively inhibited COX‐2 in vitro and in vivo. At recommended dosages by oral and SC routes in cats and dogs, robenacoxib significantly inhibited COX‐2 whilst sparing COX‐1.

### 2.2 Inhibition of pain, inflammation and fever

Robenacoxib COX inhibition is the molecular basis for suppression of pain (anti‐hyperalgesia), inflammation and fever, actions that have been demonstrated in mice, rats, dogs and cats.

#### 2.2.1 Mouse and rat

In a carrageenan‐induced rat paw oedema assay, robenacoxib dose‐dependently reduced swelling. Maximum inhibition was obtained with the 3 mg/kg dosage, for which percentage inhibitions were 78 and 77% at 3 and 5 h, respectively (King et al., 2009). In a similar model in mice, robenacoxib dose‐dependently reduced pain and swelling over the dosage range of 3.2 to 100 mg/kg SC (Beninson et al., 2018). In the Randall–Selitto assay in rats, robenacoxib exerted anti‐nociception effects at dosages of 10 and 30 mg/kg orally but not at 1 and 3 mg/kg (King et al., 2009). In a rat LPS‐induced fever model, robenacoxib and diclofenac dose‐dependently inhibited fever. The ID_50_ for robenacoxib was 1.12 mg/kg.

#### 2.2.2 Dog

**FIGURE 7:**
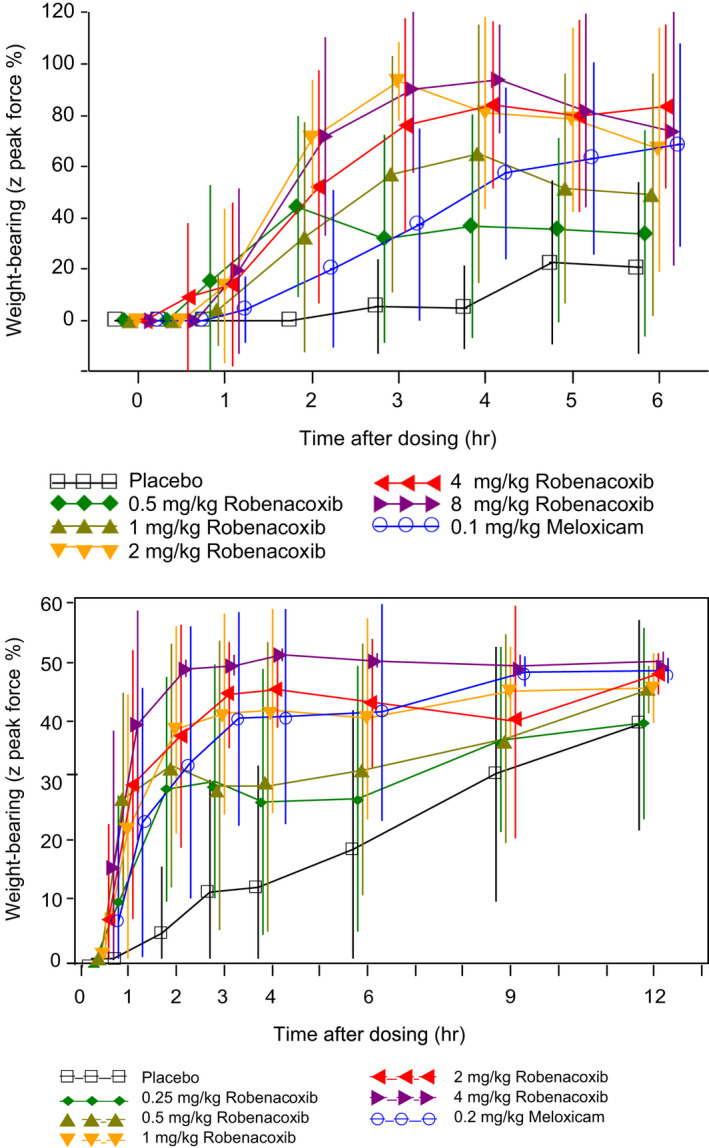
Comparison of effects of robenacoxib, meloxicam and placebo in dogs assessed by the Z peak force of a force plate. Upper: administered PO 3 h after intra‐articular injection of urate crystals (Borer et al., 2017). Lower: administered SC 3 h after intra‐articular injection of urate crystals. (Schmid et al., 2010b). Data are mean ± SD. To add clarity, symbols are distributed around the true time points

In a urate crystal model of stifle joint acute synovitis in Beagle dogs, the dose–response relationships for weight‐bearing and analgesic and anti‐inflammatory actions were established. Robenacoxib was administered once at dosages of 0.5–8 mg/kg PO (Borer et al., 2017) and 0.25–4 mg/kg SC (Schmid et al., 2010b) (Figure 7). Placebo and meloxicam were controls. The efficacy of robenacoxib was dose‐related over the range of 0.5–2 mg/kg PO and 0.25–1 mg/kg SC with a plateau response at higher dosages. The ED_50_ for improved weight‐bearing was 0.6–0.8 mg/kg PO and 0.90–1.23 mg/kg SC. Based on criteria of superior efficacy to placebo and at least equivalent efficacy to meloxicam, dosages of 2 mg/kg (SC and PO) for surgery and 1 mg/kg (PO) for osteoarthritis (OA) were selected. The onset of action of robenacoxib, administered both PO and SC, was more rapid than for meloxicam.

#### 2.2.3 Cat

**FIGURE 8:**
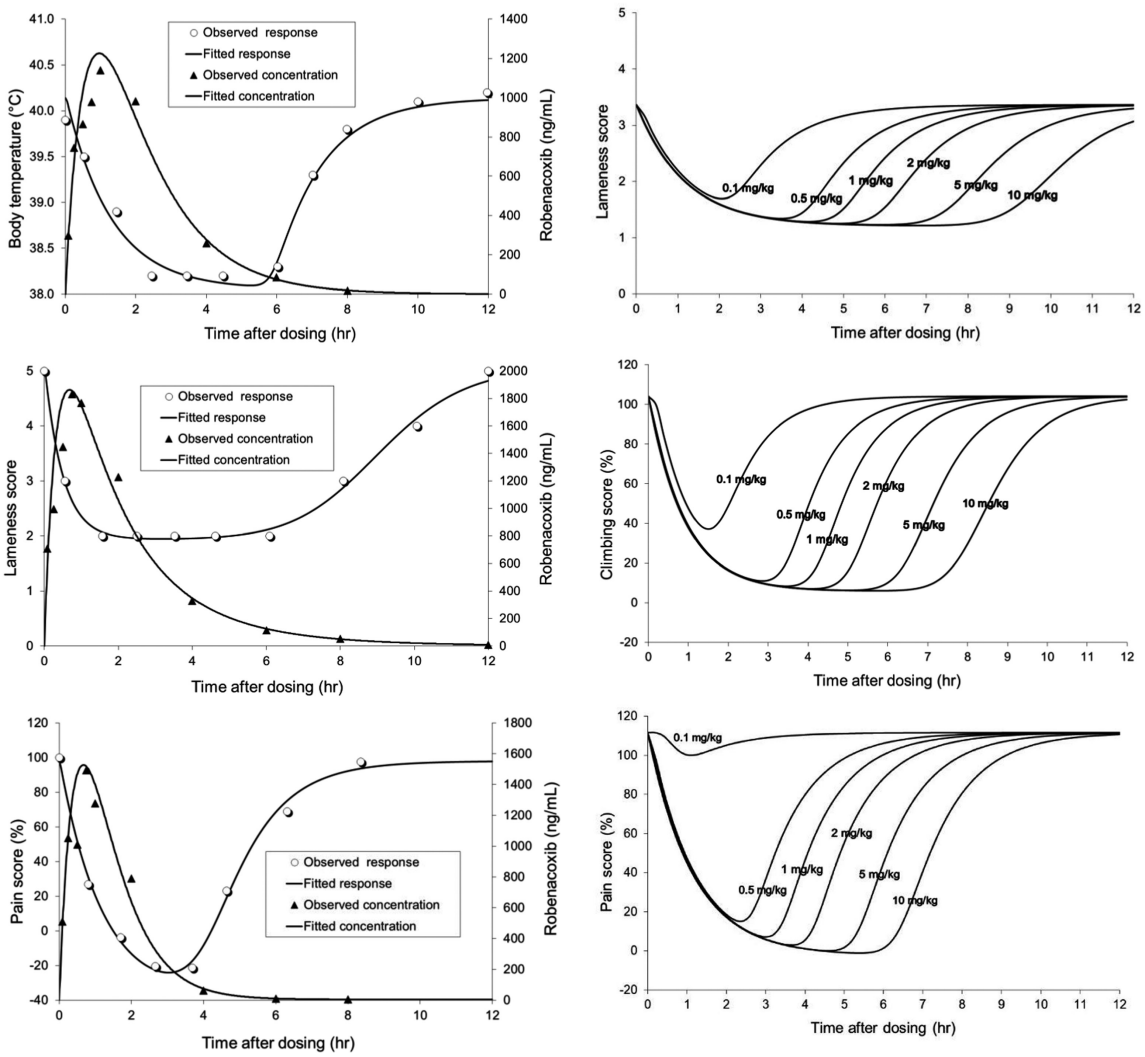
Time courses in single representative cats of observed and fitted effects and robenacoxib blood concentration (ng/ml) for body temperature, lameness score and pain score (left) and modelling of effects of different dosages for lameness, climbing and pain scores (right). The kaolin paw model was used (Giraudel et al., 2009a)

In a kaolin‐induced paw inflammation model, lameness score, locomotion, body and skin temperatures, and thermal pain threshold responded to robenacoxib (2 mg/kg SC) (Giraudel et al., 2009a). PK/PD modelling indicated a 5‐ to 7‐h duration of action (Figure 8), shorter than in the dog urate synovitis model (Schmid et al., 2010b), possibly due to a more severe challenge. Duration of action was longer in field studies in cats (King et al., 2012b; King et al., 2016).

Dosages selected by PK/PD modelling were 2 mg/kg for SC administration (surgery) and 1 mg/kg for PO dosing (acute and chronic musculoskeletal disorders and surgery).

### 2.3 Renal pharmacodynamics

NSAIDs have the potential to suppress inflammation in chronic kidney disease (CKD). However, they are also potentially nephrotoxic, for example by inhibiting vasodilation of afferent arterioles and hyperosmolality‐induced apoptosis.

Robenacoxib (30 mg/kg PO) exerted no biologically relevant effects on renal function in rats (King et al., 2009). Although serum creatinine concentrations were significantly higher with robenacoxib (0.50 mg/dl) compared with a control group (0.47 mg/dl), the effect was numerically small and there was no effect on urine creatinine and PGE_2_ concentrations, urine volume and glomerular filtration rate (GFR). In contrast, diclofenac significantly reduced urine volume and PGE_2_ concentration.

The effects of ketoprofen (COX‐1 selective) and robenacoxib (COX‐2 selective) on furosemide‐induced renal responses and COX isoform immunolocalization in the healthy cat kidney were investigated (Pelligand et al., 2015). Neither drug altered furosemide‐induced diuresis and natriuresis. It was concluded that both COX‐1 and COX‐2 generate PGs signalling macula densa renin secretion and the aldosterone response to furosemide. In addition, COX‐2 may be involved in regulating pathways other than angiotensin II‐stimulated aldosterone secretion.

Concomitant administration of an angiotensin‐converting enzyme (ACE) inhibitor and an NSAID may induce acute renal damage in humans, especially when combined with diuretics (Whelton, 1999). Nevertheless, their combined use may be appropriate, for example in animals suffering pain/inflammation and concomitant cardiovascular diseases or CKD. The effects of robenacoxib and the ACE inhibitor benazepril, alone and in combination, were therefore investigated, both with and without furosemide administration, in healthy cats (King et al., 2016b) and dogs (Panteri et al., 2017). The combination was well tolerated in both species. In cats, compared to a placebo group treated with furosemide, GFR was increased by benazepril (females only) but decreased by robenacoxib (males only). In dogs, GFR was not reduced either with or without furosemide co‐administration and urine aldosterone concentrations were significantly reduced (Panteri et al., 2017).

These findings in healthy animals did not identify increased acute kidney injury risk with combined benazepril and robenacoxib administration. However, a similar safety level might not apply in dehydrated animals or in canine and feline cardiac or kidney disease patients.

In both studies, robenacoxib (and benazepril) attenuated the furosemide‐induced increases in plasma (cat) or urine (dog) aldosterone concentrations. As aldosterone is an important mediator of the pathogenesis of some cardiovascular diseases, robenacoxib administration, alone or in combination with an ACE inhibitor, may be beneficial, for example in proteinuric CKD.

### 2.4 Other pharmacodynamic properties

Non‐selective NSAIDs suppress blood clotting by inhibiting COX‐1. Consistent with its COX‐1 sparing action, this did not occur with robenacoxib. In mice, robenacoxib did not inhibit clotting or affect haematology variables over the dosage range of 3.2–100 mg/kg SC (Beninson et al., 2018). In healthy cats and dogs, both clinical and higher robenacoxib dosages had no effect on activated partial thromboplastin, prothrombin or buccal mucosal bleeding time (BMBT) (Heit et al., 2020; King et al., 2011; King et al., 2012a; Toutain et al., 2017).

No change in BMBT was detected with robenacoxib in dogs undergoing orthopaedic or soft tissue surgery (2 mg/kg, SC) (Gruet et al., 2011, 2013) or in cats undergoing ovariectomy (1 mg/kg, PO) (Sattasathuchana et al., 2018).

Potential safety concerns of coxib NSAIDs are wound healing inhibition (including pre‐existing gastrointestinal ulcers) and increased risk of myocardial ischaemia or stroke. Signals for these effects were not detected in robenacoxib safety and clinical studies in cats and dogs (vide infra).

In common with carprofen and meloxicam, robenacoxib reduced sodium nitroprusside‐induced apoptosis in canine cruciate ligament cells (Waldherr et al., 2012), indicating a possible cytoprotective action.

Compensatory reactions of four NSAIDs (carprofen, meloxicam, indomethacin and robenacoxib) on osteogenic differentiation in canine bone marrow‐derived mesenchymal stem cells were investigated (Oh et al., 2014). Osteocalcin production was not suppressed, and PGE_2_‐related receptor and enzyme gene expression was upregulated. These findings might account for the discrepancy between the suppressant effect of NSAIDs on osteogenesis in vitro and the rarely reported deterioration of bone healing arising from NSAID clinical use.

Constitutive endothelial COX‐2 expression is protective in thromboembolic diseases, and there is concern regarding NSAID safety in human patients with ischaemic heart disease and stroke. Whilst myocardial ischaemia and stroke are rare diseases in dogs and cats, it remains to be determined whether chronic inhibition of endothelial cell COX‐2 is detrimental in age‐related diseases in these species. No signal for adverse cardiovascular effects of robenacoxib was detected in safety or clinical studies.

The viability of cultured, canine vascular endothelial cells was dose‐dependently reduced by carprofen, meloxicam and robenacoxib. Therefore, these NSAIDs might serve as adjuvant anti‐angiogenic drugs in dogs with malignant tumours (Horikirizono et al., 2019).

Robenacoxib (2 mg/kg SC in dogs) decreased the minimum alveolar concentration of the inhalation anaesthetic, sevoflurane, required to blunt the adrenergic response (MAC‐BAR). MAC‐BAR provides a quantitative measure of anaesthetic potency. There was a slight robenacoxib sparing effect on sevoflurane requirement (17%) (Tamura et al., 2014).

Robenacoxib and meloxicam did not affect insulin secretion in either conscious or anaesthetized dogs, nor did they affect amino acid infusion attenuation of decreased body temperature and heart rate in anaesthetized animals (Takashima et al., 2019).

Ketoprofen and robenacoxib very weakly manifested activation‐induced CD25 expression on murine CD4+ and CD8+ T cells in vitro (Gregorczyk & Maślanka, 2019).

## 3 PHARMACOKINETICS

### 3.1 Protein binding and distribution in blood

As with most NSAIDs, robenacoxib is highly plasma protein‐bound (King et al., 2009). At a concentration of 2000 ng/ml, protein binding exceeded 98% in the dog and cat (Jung et al., 2009). The potential for drug interactions with NSAIDs, including robenacoxib, arising from competition for plasma protein binding sites should be minimal as, at most, increased free concentrations should occur only transiently (Toutain & Bousquet‐Mélou, 2002).

Robenacoxib blood:plasma concentration ratios were 0.44:1 and 0.65:1 in the dog and cat, respectively (Jung et al., 2009). Assuming a red cell volume of ~45% in dogs and ~35% in cats, these ratios indicate that robenacoxib is present almost entirely in plasma.

### 3.2 Rat

After IV dosing in the rat, robenacoxib pharmacokinetic (PK) parameters were 2.4 ml/min/kg (plasma clearance), 306 ml/kg (volume of distribution at steady state) and 1.9 h (terminal *T*_1/2_). After oral dosing, PK variables were 1 h (T_max_), 1.3 h (terminal *T*_1/2_) and 80% (bioavailability) (King et al., 2009).

### 3.3 Dog

Robenacoxib PK data after IV and SC injection and oral dosing in Beagle dogs, both with and without feed, are presented in Table 8 (Jung et al., 2009). Inter‐animal variability was relatively low.

**TABLE 8:** Pharmacokinetic parameters and variables for robenacoxib in dogs

Parameter or variable (units)	Administration route			
	Intravenous (IV)	Subcutaneous (SC)	Oral, fasted	Oral, fed
T_max_ ^a^ (h)	_	0.50	0.50	0.25
C_max_ ^b^, ^c^ (ng/ml)	5531 ± 895	657 ± 195	947 ± 515	832 ± 397
AUC_(0‐inf)_ ^b^, ^c^ (ng h/ml)	1235 ± 259	1090 ± 134	1023 ± 203	782 ± 139
MRT^b^ (h)	0.30 ± 0.04	1.20 ± 0.27	1.00 ± 0.47	0.94 ± 0.48
MAT^b^ (h)	–	0.90 ± 0.26	0.67 ± 0.45	0.59 ± 0.49
*T_½_* (h)^d^	0.63 ± 0.20	0.81 ± 0.19	0.81 ± 0.27	1.01 ± 0.71
Cl (L/kg/h)	0.81 ± 0.19			
Vc (L/kg)	0.18 ± 0.03			
Vss (L/kg)	0.24 ± 0.04			
Vd_area_ (L/kg)	0.77 ± 0.17			
Bioavailability	1	0.88 ± 0.15	0.84 ± 0.19	0.62 ± 0.09

Note

T_max_, time of maximum blood concentration; C_max_, maximum blood concentration; AUC_(0‐inf)_, area under the blood concentration–time curve to infinity; MRT, mean residence time; MAT, mean absorption time; *T*_½_, terminal half‐life; Cl, dosage/AUC_(0‐inf)_; Vc, volume of central compartment; Vd_area_, apparent volume of distribution in elimination phase; Vss, apparent volume of distribution at steady state; bioavailability (F), AUC_(0‐inf)_ after oral or SC dosing/⁄AUC_(0‐inf)_ after IV dosing.

Data are mean ± SD (*n* = 12) (Jung et al., 2009). Dosage = 1 mg/kg (exact for IV and SC dosing, nominal for oral).

^a^ Median.

^b^ Geometric mean.

^c^ Normalized by individual dosage for oral administration (calculated for dosage of 1.0 mg/kg for all animals).

^d^ Harmonic mean.

Robenacoxib body clearance was moderate (0.81 L/kg/h) and steady‐state distribution volume relatively low (240 ml/kg). T_max_ was rapidly attained after both oral and SC administration (0.25–0.5 h), correlating with the rapid onset of action reported in dog urate synovitis studies (Borer et al., 2017; Schmid et al., 2010b). Absolute bioavailability was 88% after SC injection, 84% after oral administration in fasted dogs and 62% in fed dogs. Robenacoxib PK did not deviate from linearity over the dosages tested: 2–10 mg/kg PO (King et al., 2011), 0.25–4 mg/kg SC (Schmid et al., 2010b) and 0.5–8 mg/kg PO (Borer et al., 2017) (Table 9).

**TABLE 9:** Blood pharmacokinetic variables for robenacoxib administered orally once daily for 6 months at four dosages in dogs

Dose (mg/kg)	Month	AUC/dosage (ng h/ml)/(mg/kg/day)^a^		Cmax/dosage (ng/ml)/(mg/kg/day)^a^	
		Geometric mean	CV (%)	Geometric mean	CV (%)
2	0	917	35	549	48
	6	1145	32	500	77
4	0	797	33	330	57
	6	1074	32	421	78
6	0	859	37	449	64
	6	1027	43	340	81
10	0	1017	36	491	46
	6	936	31	410	95

^a^ Mean values normalized for dosage of 1 mg/kg (King et al., 2011). CV, coefficient of variation.

**FIGURE 9:**
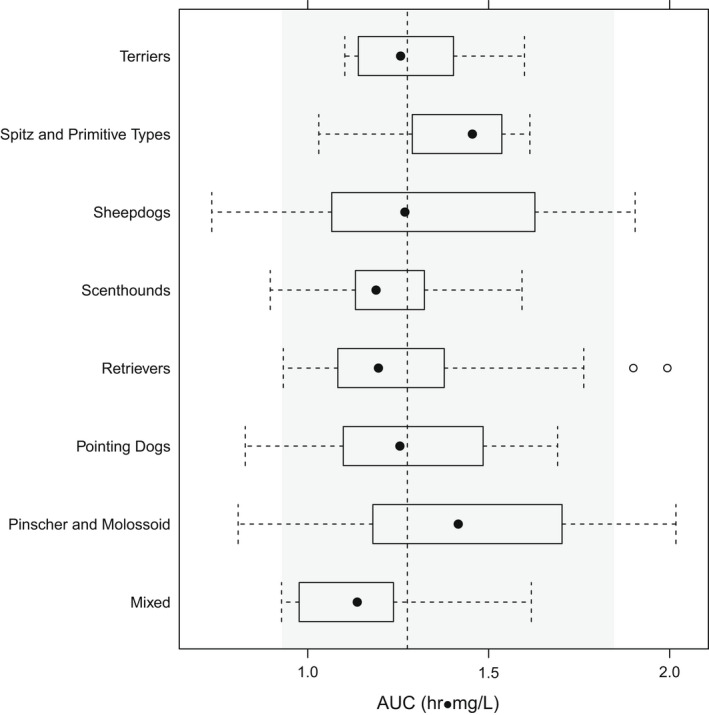
Variability in robenacoxib exposure (indicated by AUC_(0‐inf)_) for grouped breeds of dogs with OA. The vertical dashed line denotes the median AUC (1.28 mg h/ml), and the grey area denotes the range between 5th and 95th percentiles. There were no significant differences (*p* > .01) between breed groups (Fink et al., 2013)

**FIGURE 10:**
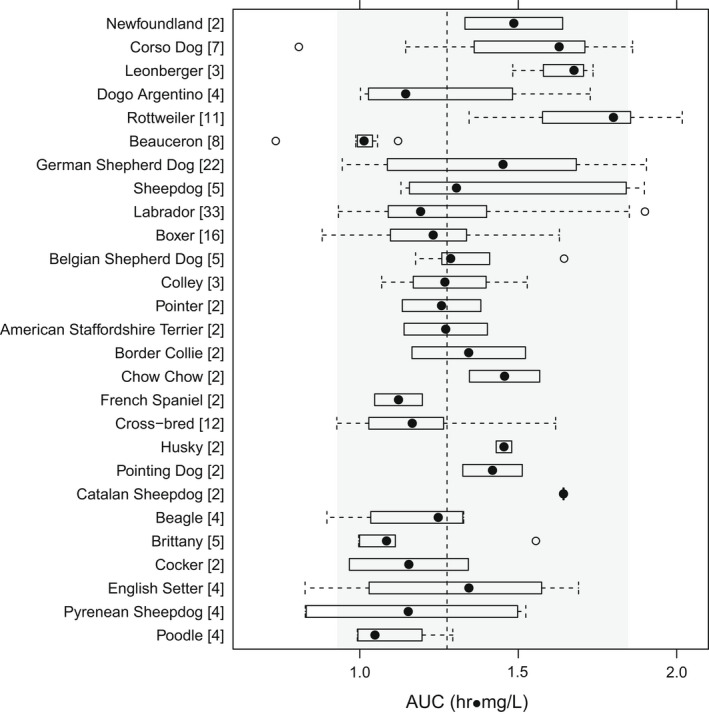
Variability in robenacoxib exposure (assessed from AUC_(0‐inf)_ for individual breeds (*n* ≥ 2 animals)) after PO administration to dogs with OA. The dashed line denotes the median AUC (1.28 mg h/ml), and the grey area denotes the range between 5th and 95th percentiles. There were no significant differences between breeds (*p* > 0.01) (Fink et al., 2013)

Celecoxib, cimicoxib and mavacoxib display pharmacogenetic polymorphism and metabolic variability, both within and between dog breeds, which impacts on clinical use (Cox et al., 2011; Jeunesse et al., 2013; Martinez et al., 2013; Paulson et al., 1999). No such evidence has been reported for robenacoxib. A population PK study, in a clinical cohort of 208 OA dogs of 62 breeds, demonstrated that body clearance of robenacoxib did not differ, and therefore, no dose adjustment was needed for age, sex, breed or breed group (Figures 9, 10) (Fink et al., 2013).

The population PK profile of robenacoxib in blood and stifle joint synovial fluid was investigated in eight Beagle dogs with urate‐induced stifle inflammation, and in a cohort of 95 dogs with a clinical diagnosis of OA (Silber et al., 2010). Clearance in healthy Beagle dogs was 75% higher than in OA dogs. Possible causes of this difference include the slightly greater age and lower average weight of OA dogs, and inhibition of cytochrome P450 (CYP) by chronic inflammation in OA. Inflammatory mediators (e.g. cytokines) affect the activities and levels of CYP and other drug‐metabolizing enzymes (Renton, 2001; Renton, 2005). Likewise, a longer elimination T_1/2_ was reported for mavacoxib in OA dogs compared with healthy young Beagles (Cox et al., 2011).

The residence time for robenacoxib in inflamed joints was longer than in blood in both healthy and OA dogs. Concentrations exceeded the IC_50_ for COX‐2 for 16 h (OA dogs) and 10 h (healthy dogs) at a dosage of 1.5 mg/kg.

Anaesthesia may alter robenacoxib's PK profile; in Beagles, Cmax was lower (1.3 vs. 2.2 µg/ml) and T_max_ delayed (120 vs. 90 min) in sevoflurane‐anaesthetized compared with conscious dogs (Oyama et al., 2018).

### 3.4 Cat

Cat PK data for robenacoxib after single IV, SC and oral administrations are presented in Table 10 (King et al., 2013). T_max_ (oral 0.5 h, SC 1 h) was short. The volume of distribution at steady state (190 ml/kg) and body clearance (0.44 L/kg/h) were relatively low.

**TABLE 10:** Pharmacokinetic parameters and variables for robenacoxib in cats

Variable or parameter	Route of administration							
	Intravenous (IV)		Subcutaneous (SC)		Oral. Fed entire daily ration		Oral. Food withheld	
	Geo mean	CV (%)	Geo mean	CV (%)	Geo mean	CV (%)	Geo Mean	CV (%)
C_max_ or C_0_ ^a^, ^b^ (ng/ml)	7365	25	732	21	125	116	773	52
AUC_(0‐tlast)_ ^b^ (ng h/ml)	2276	21	1554	17	207	91	1090	37
AUC_(0‐inf)_ ^b^ (ng h/ml)	2282	21	1564	17	225^b^	90	1122	35
MRT (h)	0.44	25	1.67	26	NC	NC	1.48	65
*T_½_* (h)	1.49	28	1.11	13	NC	NC	1.71	36
Clearance Blood^c^ (L/kg/h)	0.44	20	—	—	—	—	—	—
Clearance Plasma^c^ (L/kg/h)	0.29	20	—	—	—	—	—	—
Vd_area_ Blood^c^ (L/kg)	0.94	27	—	—	—	—	—	—
Vd_area_ Plasma^c^ (L/kg)	0.61	27	—	—	—	—	—	—
Vss Blood^c^ (L/kg)	0.19	31	—	—	—	—	—	—
Vss Plasma^c^ (L/kg)	0.13	31	—	—	—	—	—	—
Bioavailability (%)^b^	—	—	0.69	13	0.10	88	0.49	31

Note

Data are from 12 cats (King et al., 2013). Geo mean: geometric mean; dosage of robenacoxib = 2 mg/kg IV and SC and 6 mg per animal oral administration, with two feeding schedules.

Abbreviations: CV, coefficient of variation; NC, not calculated.

^a^ For IV administration, concentration is by extrapolation to time 0 (C_0_).

^b^ Data normalized to a dosage of 1 mg/kg.

^c^ Clearance and volumes of distribution determined from measured concentrations in blood.

Similar results were reported in other feline studies: volume of distribution at steady state (respectively 0.20, 0.19 and 0.21 L/kg) and clearance (0.63, 0.54 and 0.502 L/kg/h) (Giraudel et al., 2009a; Pelligand et al., 2012, 2016).

Robenacoxib absolute bioavailability in fasted cats was 69% after SC injection and 49% after oral administration without food (King et al., 2013). In one study, feed consumption (the entire daily ration offered once daily) led to a bioavailability relative to fasted cats of only 20.4%. A smaller impact of feeding occurred in a second study; the bioavailability relative to fasted cats was 104% when administered with one‐third of the daily ration and 80% with the entire daily ration. Based on PK/PD modelling of the magnitude of COX‐2 inhibition and a decreased bioavailability when the drug is given with the entire daily ration, it was recommended, for optimal efficacy, that robenacoxib tablets should be administered either without or with a small amount of food. Nevertheless, administration with food may provide appropriate concentrations for chronic conditions, for example OA in cats (King et al., 2013).

A population PK analysis was conducted using data from several studies after SC and IV administration: 47 densely sampled laboratory cats and 36 clinical cats sparsely sampled peri‐operatively. There was no effect of age, body weight and sex (as for dogs) or anaesthesia on robenacoxib clearance (Fink et al., 2013; Oyama et al., 2018; Pelligand et al., 2016).

There are no published robenacoxib PK dose‐linearity studies in cats.

Low robenacoxib concentrations were detected in aqueous humour after oral dosing, indicating that the drug crosses the intact blood–aqueous barrier (Sharpe et al., 2018).

### 3.5 Species comparison of pharmacokinetics

In the rat, dog and cat, T_max_ was relatively short after oral administration of robenacoxib. The data are consistent with rapid absorption, but the conclusion is not definitive because a short T_max_ could be due to flip‐flop PK. Consistent with the short T_max_, oral robenacoxib had a relatively rapid onset of action in the dog urate synovitis model (Borer et al., 2017).

Orally administered robenacoxib should be rapidly absorbed from the small intestine, given its relatively high aqueous solubility of 0.17 g/L at pH 6.8. Moreover, its medium lipid solubility (log partition coefficient in n‐octanol/phosphate buffer at pH 6.8 = 2.27) facilitates intestinal absorption (King et al., 2009). The absorption from the stomach is limited or absent, a consequence of its poor solubility in acidic conditions (water solubility at pH 3 < 0.01 g/L) even though it is non‐ionized at this pH (pKa = 4.7).

The steady‐state distribution volume of robenacoxib is low (240 ml/kg in dogs; 190 ml/kg in cats) but greater than blood volume (70–90 ml/kg). This is consistent with most drug remaining in the extracellular compartment. More precisely, using the Oie and Tozer model (Oie & Tozer, 1979), which predicts drug repartition in human body fluids, it has been computed, for a degree of binding of 98%, that is an fu = 0.02 and Vss of 0.24 L/kg (dogs) or 0.19 L/kg (cats), that the percentage of robenacoxib remaining in extracellular fluids is approximately 50% in dogs and 67% in cats (Table 11, 12). For firocoxib with fu = 0.03 and a larger steady‐state volume of distribution of 2.6 L/kg (McCann et al., 2004), the corresponding value is 4% (Table 11). Pharmacological, toxicological and clinical implications of such differences remain unknown, even though it is generally accepted that intracellular drug concentrations are important to drug efficacy and toxicity and also to predict drug interactions and inter‐subject variability in drug response (either on‐target or off‐target effects) (Chu et al., 2013).

**TABLE 11:** Repartition in body fluid compartments of robenacoxib and firocoxib in dogs based on the Oie & Tozer model (Oie & Tozer, 1979)

	PK parameter	Location of robenacoxib/firocoxib in the body (%)			
		Plasma	Interstitial fluid	Plasma and interstitial fluid	Intracellular fluid
Robenacoxib		20.8	32.3	53.1	46.9
Vss (L/kg)	0.24				
fu	0.02				
Firocoxib		1.72	2.72	4.44	95.6
Vss (L/kg)	2.9				
fu	0.03				

Note

The model predicts that most firocoxib (large volume of distribution) is located in intracellular fluids (96%), whereas robenacoxib (small volume of distribution) is evenly divided between intra‐ and extracellular fluids (47 vs 53%).

**TABLE 12:** Concentrations of robenacoxib and firocoxib unbound and bound in dog body fluid compartments based on the Oie & Tozer model (Oie & Tozer, 1979)

	PK parameter	Percentage of drug unbound vs. bound to proteins located in:			
			Plasma	Extracellular fluid	Intracellular fluid
Robenacoxib		Unbound	0.417	1.67	3.75
Vss (L/kg)	0.24	Bound	20.42	30.62	43.12
fu	0.02				
Firocoxib		Unbound	0.0517	0.206	0.466
Vss (L/kg)	2.9	Bound	1.672	2.5	95.1
fu	0.03				

Note

The model predicts total body fractions for robenacoxib: unbound = 5.83%, bound = 94.2%. Total body fractions for firocoxib: unbound = 0.72%, bound = 99.3%. The amount of free robenacoxib (active fraction) in the extracellular compartment (biophase) (1.67%) is higher than for firocoxib (0.206%).

The body clearance of robenacoxib was moderate (0.81 L/kg/h) in dogs and low (0.44 L/kg/h) in cats. The difference might reflect reduced glucuronide conjugation capacity in the cat (van Beusekom et al., 2014).

Robenacoxib clearance is consistent with low hepatic extraction in both species. Therefore, hepatic blood flow may not be the limiting factor in robenacoxib clearance. Assuming virtually no renal clearance, the maximal hepatic extraction ratio would be approximately 0.13 and 0.05, respectively, for dogs and cats, indicating a maximal possible oral bioavailability of 87% in dogs and 95% in cats. Therefore, the reduced oral bioavailability of robenacoxib in dogs and cats, when administered with a large amount of food, is not primarily caused by first‐pass metabolism. Many factors can influence rate and extent of drug absorption from the gastrointestinal tract, including sequestration in digesta (Toutain & Bousquet‐Mélou, 2004).

The excretion of IV administered ^14^C‐radiolabelled robenacoxib was primarily in faeces, 64.6% in dogs and 72.5% in cats, consistent with elimination in bile following hepatic metabolism (King & Jung, 2021).

Robenacoxib metabolites and roles of specific cytochrome P450 enzymes in their formation have not been determined. Speculatively, however, robenacoxib metabolism is likely to be similar to that of its analogue, lumiracoxib, which, in humans, undergoes extensive hepatic metabolism, primarily by oxidation and hydroxylation with additional glucuronidation (Rordorf et al., 2005).

Renal clearance of unchanged robenacoxib should be low because glomerular ultrafiltration will be limited by high plasma protein binding. Furthermore, tubular reabsorption of robenacoxib should be relatively high; cat and dog urines are normally more acidic (pH ~6) than blood (~7.4), which favours passive reabsorption (King et al., 2009). The renal clearance of radiolabelled compound may therefore be predominantly as polar metabolites (King & Jung, 2021).

### 3.6 Pharmacokinetics — effects of kidney and liver disease and drug interactions

The elimination of radiolabelled robenacoxib was primarily hepatic in dogs (65%) and cats (72%) (King & Jung, 2021). From the minor contribution of urinary excretion to elimination, there should be, at most, a requirement for minor dose adjustment in animals with renal insufficiency. Dose adjustment might be necessary, however, in animals with severe liver disease. The clinical use of NSAIDs as a group should be undertaken with caution, even contraindicated in such cases, because of the risk of hepatotoxicity. However, the term liver disease covers a wide spectrum of conditions, and not all are associated with hepatotoxic actions of NSAIDs.

A population PK analysis in OA dogs indicated no significant effect on robenacoxib exposure of kidney and liver variables (plasma concentrations of total protein, urea and creatinine, and activities of aspartate aminotransferase, alanine aminotransferase and gamma‐glutamyltransferase (Fink et al., 2013)).

Robenacoxib PK interactions with drugs of other classes have not been specifically investigated, although no adverse interactions have been reported in field studies.

### 3.7 Pharmacokinetics in inflammatory exudate and synovial fluid

Those NSAIDs that are acids tend to concentrate in and persist at sites of inflammation. This property was termed ‘tissue selectivity’ (Brune & Furst, 2007), although ‘selectivity for inflammatory sites’ might be more appropriate.

The selective distribution of robenacoxib to sites of inflammation has been demonstrated in rats, dogs and cats, and is attributable to its physicochemical property as a weak acid (pKa 4.7) and high degree of plasma protein binding. A long residence time of robenacoxib in exudate was demonstrated in subcutaneously implanted tissue cages, when acute inflammation was induced by zymosan (rats) (King et al., 2009) or carrageenan (cats) (Pelligand et al., 2012; Pelligand et al., 2014).

In rats, the AUC_(0‐inf)_ of robenacoxib (2 mg/kg PO) was 2.9 times higher in inflammatory exudate than in blood, and MRT was three times longer (King et al., 2009). Robenacoxib inhibited COX‐2 at all time points measured (vide supra).

After IV, SC and oral robenacoxib administration (2 mg/kg) in cats, the mean residence time (MRT) was short (0.4, 1.7–1.9 and 3.2 h, respectively) (Table 13). In tissue cage inflammatory exudate, MRT was approximately 24 h for all routes of administration. As in rats, exudate concentrations of robenacoxib inhibited COX‐2 over 24 h in cats (Pelligand et al., 2012, 2014).

**TABLE 13:** Blood and exudate pharmacokinetic parameters and variables for robenacoxib in cats

Variables and parameters	Biological matrix	IV 2 mg/kg	SC 2 mg/kg	Oral 6 mg tablet	SC 2 mg/kg
		Pelligand et al. (2012)			Pelligand et al. (2014)
C_0_ or C_max_ ^a^ (ng/ml)	Blood	9001	1905	794	1313
	Exudate	31.5	39.9	17.9	85.2
T_max_ ^b^ (h)	Blood	NA	0.60	1.64	0.90
	Exudate	4.40	7.10	9.60	8.10
AUC_(0‐last)_ ^a^ (dose normalized) (ng h/L)	Blood	1864	1861	958	3043
	Exudate	1123	1235	720	ND
MRT^b^ (h)	Blood	0.361	1.68	3.22	1.85
	Exudate	25.9	23.3	23.5	ND
*T_½_* ^c^ (h)	Blood	0.843	1.04	0.78	1.13
	Exudate	21.7	12.2	16.2	ND

Note

IV, intravenous; SC, subcutaneous; AUCs were normalized to 1 mg/kg dosage in Pelligand et al. (2012) but not in Pelligand et al. (2014); ND, not determined.

^a^ Geometric mean. C_0_ extrapolated to time 0.

^b^ Arithmetic mean.

^c^ Harmonic mean.

^d^ Inflammatory exudate induced by intra‐caveal injection of 1 ml sterile carrageenan 2% solution.

In dogs, robenacoxib residence time in inflamed stifle joint synovial fluid (urate crystal‐induced synovitis and naturally occurring OA) was longer than in synovial fluid from non‐inflamed joints or blood (Silber et al., 2010).

The selectivity of robenacoxib for inflammatory sites contributes to a longer duration of action in disorders associated with peripheral inflammation than predicted from its short blood half‐life. Robenacoxib reduced pain and inflammation in both experimental models and clinical cases of musculoskeletal disorders (acute and chronic, including degenerative joint disease (DJD) and OA), and surgery with once‐daily dosing (see Section 5, Efficacy and Safety in Clinical Use).

In contrast, the duration of action of robenacoxib in non‐inflammatory biophases is predicted to be shorter. For example, in fever in cats, with an assumed site of action in the hypothalamus, the duration of action in attenuating hyperthermia was approximately 6 h (Giraudel et al., 2009a). The duration of anti‐pyretic action of robenacoxib in clinical cases is not known but is likely to be short.

In conditions involving pain and inflammation, the action of robenacoxib is predicted to be persistent only if inflammation is present. Otherwise, the drug will be eliminated rapidly from the central compartment. For example, in the canine urate synovitis model, firocoxib (plasma *T*_1/2_ ~5.9 h) (McCann et al., 2004) was effective when dosed 13 h prior to challenge but robenacoxib (blood T_1/2_ ~1 h) was not (Dauteloup et al., 2017). Robenacoxib was effective in the same model, however, when administered after intra‐synovial urate injection (Borer et al., 2017; Schmid et al., 2010b). The implication for clinical use is that robenacoxib should be administered shortly before elective surgery in healthy animals, for example for ovariohysterectomy.

## 4 SAFETY: PRE‐CLINICAL STUDIES

### 4.1 Rat

In rats, the gastric and intestinal tolerability of robenacoxib was greater than that of diclofenac. The data correlated with COX‐1 inhibition by diclofenac but not by robenacoxib (see Section 2.1, Pharmacodynamics (King et al., 2009)). The mean ± SD number of gastric ulcers was 0 (vehicle control), 1.3 ± 1.8 (robenacoxib at 100 mg/kg/day) and 18.7 ± 6.6 (diclofenac 100 mg/kg/day). Robenacoxib (10, 30 and 100 mg/kg over 4 days) increased intestinal permeability to a lesser degree than 10 mg/kg diclofenac.

Robenacoxib had no toxicologically relevant renal effects at a dosage of 30 mg/kg (see Section 2.3, Pharmacodynamics (King et al., 2009)).

### 4.2 Dog

In Beagle dogs, robenacoxib administered orally once daily, at dosages of 10, 20 and 40 mg/kg for one month and 0, 2, 4, 6 and 10 mg/kg for 6 months, produced no significant adverse effects, based on clinical observations, haematological and clinical chemistry variables, and the absence of macroscopic and microscopic lesions at necropsy (King et al., 2011). In the 6‐month study, there were no adverse effects on BMBT and stifle joint tissues, electrocardiographic and ophthalmoscopic examinations, and urinalysis. The highest dosages administered correspond to 20–40 (one month) and 5–10 (6 months) multiples of the clinical robenacoxib PO dosage for long‐term use (OA).

These findings were confirmed after administration of the flavoured tablet formulation of robenacoxib (0, 2, 6, and 10 mg/kg once daily for six months) to Beagle dogs. There were no demonstrable adverse effects on body weight, feed and water consumption, urinalysis, faecal examination and stifle joint tissues (Toutain et al., 2018). Clinical pathology data indicated only increased eosinophil count (10 mg/kg) and reduced ovary weight (6 and 10 mg/kg). Histopathology of all tissues/organs was normal.

Single robenacoxib doses (2 and 4 mg/kg IV and 2 mg/kg SC) exerted no significant effects on arterial blood pressure, heart rate, electrocardiogram (ECG), body temperature, BMBT, blood haematology, coagulation and clinical chemistry variables (Desevaux et al., 2017).

To support interchangeable use of injectable and tablet formulations, a safety study was conducted in cross‐bred hound dogs administered 2, 4 and 6 mg/kg robenacoxib, with three 20‐day treatment cycles, separated by 14‐day wash‐out periods (Toutain et al., 2017). There were no robenacoxib formulation‐related changes in body weight, food consumption, ophthalmic and neurological examinations, electrocardiogram, BMBT, clinical pathology and organ weights. Treatment‐related differences, of low incidence at all dosages, comprised macroscopic and microscopic changes at injection sites and microscopic gastrointestinal tract findings.

### 4.3 Cat

Robenacoxib administration PO to cats (5 and 10 mg/kg once daily for 28 days and 2, 6 and 10 mg/kg twice daily for 42 days) produced no toxicological effects based on general health, haematological and clinical chemistry variables; urinalyses; and organ weight, gross pathology and histopathology (King et al., 2012a).

Single‐dose robenacoxib administration, IV (2.0 and 4.0 mg/kg) and SC (2 mg/kg), was well tolerated in healthy cats (Panteri et al., 2017).

To support interchangeable use of injectable and tablet formulations, cats were administered robenacoxib at 2, 4 and 6 mg/kg (SC) and 2.4, 4.8 and 7.2 mg/kg (PO) (Heit et al., 2020). Ten‐day treatment cycles comprised seven days of oral followed by three days of SC administration, once daily and, after the third cycle, an additional seven‐day oral dose (total of 37 days). All cats remained in good health. There were no changes in body weight and food consumption and no ophthalmic, physical or neurological adverse effects. Treatment‐related abnormalities were of low occurrence, comprising transient oedema with mild, subacute/chronic inflammation at injection sites and QT prolongation on ECG. No adverse effects were attributable to interchanging administration route.

### 4.4 Discussion of safety

Pre‐clinical safety studies indicated that robenacoxib produces minimal adverse effects, even at high dosages, in healthy rats, dogs and cats. In rats, the tolerability of robenacoxib, including effects on the gastrointestinal tract and kidney, was greater than for diclofenac (King et al., 2009). Even low dosages of diclofenac (1 mg/kg/day PO for 28 days) induced severe gastrointestinal ulceration and nephrotoxicity in Beagle dogs (Anonymous, 2003).

As well as inhibition of the COX‐1 gastroprotective effect, NSAID‐induced gastrointestinal ulceration might be due to a direct topical action, attributable to their hydrophobic and acidic properties, disrupting the protective gastric mucus layer and exposing underlying epithelial cells to acidic gastric secretions (Lichtenberger et al., 1995; Smale & Bjarnason, 2003). In addition, acidic NSAIDs will concentrate within gastric cells via ion trapping, as they are non‐ionized in the acidic environment of the gastric lumen but ionized in the pH neutral cytoplasm of cells, where they might uncouple mitochondrial oxidative phosphorylation (Krause et al., 2003). However, neither of these hypothetical mechanisms can explain the observed difference in safety in dogs between robenacoxib and diclofenac, as both have very similar hydrophobic (water/octanol partitioning) and acidic (pKa) properties (King et al., 2009). The greater safety of robenacoxib compared with diclofenac in healthy animals can therefore be best explained by less and shorter lasting COX‐1 inhibition.

Despite its high COX‐2 selectivity, robenacoxib is predicted to inhibit COX‐1 and COX‐2 at the high dosages used in safety studies. A likely explanation for the lack of detectable robenacoxib toxicity at these dosages is short exposure time in the central compartment, leading to a relatively short duration of COX‐1 or COX‐2 inhibition in highly perfused organs, including the gastrointestinal tract and kidney (Brune & Furst, 2007; King et al., 2011; King et al., 2012a).

In dogs at the highest robenacoxib dosage tested, 40 mg/kg, the predicted maximum COX‐1 inhibition was 50% and of short duration, whilst respective values for COX‐2 were maximum inhibition of 100% for up to 10 h (Figure 5) (King et al., 2011). At the 10 mg/kg dosage in cats, the predicted maximum COX‐1 inhibition was 58% and of short duration, whilst the maximum COX‐2 inhibition was 99% (King et al., 2012a).

These pre‐clinical animal safety studies have limitations. First, only one dog breed (Beagle) was used in most studies. Feline studies were restricted to domestic short‐hair cats. It is moreover likely that most animals were highly inbred. The studies therefore have a low probability of detecting rare adverse events, including idiosyncratic liver toxicity, which has been reported with robenacoxib in dogs (Reymond et al., 2012). Second, the healthy young animals used are likely to have had low levels of induced COX‐2. Therefore, the studies may evaluate, primarily or solely, the effect of robenacoxib on constitutive COX of both isoforms, with a low predictive value for clinical usage, particularly in animals undergoing wound repair or having pre‐existing gastrointestinal ulcers (Wallace & Devchand, 2005) or with compromised kidney function (Cheng & Harris, 2004). No specific safety studies and relatively few PD studies have been reported on wound healing. For the kidney, no deterioration in renal function occurred when robenacoxib was co‐administered with an ACE inhibitor and furosemide in cats (King et al., 2016b) and dogs (Panteri et al., 2017).

No safety signals for robenacoxib were detected in safety and clinical studies in cats and dogs for wound healing, the gastrointestinal tract and kidney tissues (vide infra). In clinical trials, no issues related to healing were reported when robenacoxib was administered up to 15 days in dogs and cats undergoing fracture repair or up to 14 days in dogs undergoing gastrointestinal surgery (Gruet et al., 2011, 2013; Speranza et al., 2015).

## 5 EFFICACY AND SAFETY IN CLINICAL USE

Clinical studies with robenacoxib, in four categories, are summarized in Tables 14, 15, 16, 17. These comprise the following: (1) four USA placebo‐controlled studies of peri‐operative use; (2) five EU and three Japanese non‐inferiority comparison studies with a positive control; (3) clinical safety and pilot efficacy studies in cats with chronic musculoskeletal disorders (CMSD), DJD or OA; and (4) one open‐label study and six studies comparing robenacoxib with a positive control. Studies 1 to 3 were conducted by the sponsor company. Group 4 studies were conducted independently of the sponsor company.

**TABLE 14:** Summary of clinical trials with robenacoxib in dogs undergoing surgery

Indication	Country	Dosage regimen	Number of dogs^a^		Primary endpoint (efficacy)		References
			Robenacoxib	Control	Variable	Principal results^b^	
Soft tissue surgery	USA	SC injection pre‐op and for 2 days post‐op	159	158 (placebo)	Treatment success^c^	Robenacoxib 108/151 success (73.7%) vs placebo 85/152 (58.1%) *p* = .006	Friton et al. (2017a)
Soft tissue surgery	USA	Oral pre‐op and for 2 days post‐op	116	115 (placebo)	Treatment success^c^	Robenacoxib 89/116 success (76.7%) vs placebo 74/114 (64.3%) *p* = .019	Friton et al. (2017b)
Soft tissue surgery	France and Germany	Single SC injection pre‐op. Oral days 2–15 post‐op	118	56 (meloxicam)	GPS	Relative efficacy of robenacoxib/meloxicam 1.12 (0.97–1.29) *p* = .12	Gruet et al. (2013)
Orthopaedic surgery	France and Germany	Single SC injection pre‐op. Oral days 2–14 post‐op	97	43 (meloxicam)	GPS	Relative efficacy of robenacoxib/meloxicam 1.16 (0.98–1.29) *p* = .09	Gruet et al. (2011)
Laparoscopic ovariectomy and gastropexy	Italy	Single SC injection pre‐op	13	13 (meloxicam)	Not defined	GPS higher with robenacoxib at 24 h (*p* = .004) but no difference at 1, 6, 12 and 18 h	Bendinelli et al. (2019)
Laparoscopic ovariectomy	Italy	Single SC injection pre‐op	5	5 (parecoxib) 5 (morphine)	Not defined	Rescue therapy needed for morphine but not NSAIDs	Giorgi et al. (2012) (abstract)

Note

Studies are listed in decreasing order of sample size.

GPS, Glasgow pain scale. Note that different variants of this scale were used in various studies.

^a^ Maximum number of dogs included in analysis, for example for demographic or safety sample. Numbers were sometimes lower for certain analyses, for example efficacy or per‐protocol sample.

^b^ For Friton et al. (2017a, 2017b,2017a, 2017b), results are the number (%) of cases rated as treatment success in each group plus the *p* value for comparison of robenacoxib with placebo. For Gruet et al. (2011, 2013), which were non‐inferiority studies, results are the relative efficacy of robenacoxib/meloxicam (95% confidence interval) plus the *p* value.

^c^ Treatment success was defined as no requirement for rescue analgesia (assessed from the GPS) and no withdrawal from the study due to an adverse event.

**TABLE 15:** Summary of clinical trials with robenacoxib in dogs with osteoarthritis

Indication	Country	Dosage regimen	Number of dogs^a^		Primary endpoint (efficacy)		References
			Robenacoxib	Control	Variable	Main results^b^	
Osteoarthritis	France	Oral for up to 84 days	125	63 (carprofen)	Global functional disability	Relative efficacy of robenacoxib/carprofen 0.955 (0.805–1.12) *p* = .58	Reymond et al. (2012)
Osteoarthritis	Italy	Oral for 30 days	30	30 (undenaturated type II collagen)	LOAD score	No significant difference between groups (*p* = .42) in improvement	Stabile et al. (2019)
Osteoarthritis (stifle)	UK	Oral for 28 days	34	None	Not defined	Reduced lameness (*p* < .01) and synovial C‐reactive protein (*p* < .05) from baseline	Bennett et al. (2013)
Osteoarthritis	Japan	Oral for up to 28 days	21	11 (carprofen)	Global functional disability	Relative efficacy of robenacoxib/carprofen 1.24 (0.555–2.49) *p* = .25	Edamura et al. (2012)

Note

Studies are listed in decreasing order of sample size.

^a^ Maximum number of dogs included in analysis. Numbers were lower in the efficacy analysis in Stabile et al. (2019).

^b^ Reymond et al. (2012) and Edamura et al. (2012) were non‐inferiority studies, results are the relative efficacy of robenacoxib/carprofen (95% confidence interval) plus the P value.

**TABLE 16:** Summary of clinical trials with robenacoxib in cats undergoing surgery

Indication	Country	Dosage regimen	Number of cats^a^		Primary endpoint (efficacy)		Reference
			Robenacoxib	Control	Variable	Results^b^	
Onychectomy plus neuter surgery	USA	SC injection pre‐op and for 2 days post‐op	174	175 (placebo)	Treatment success^c^	Robenacoxib 139/174 success (80.3%) vs placebo 102/175 (58.3%) *p* = .037	King et al. (2016c)
Onychectomy plus neuter surgery	USA	Oral pre‐op and for 2 days post‐op	167	82 (placebo)	Treatment success^c^	Robenacoxib 137/167 success (73.5%) vs placebo 43/82 (53.7%) *p* = .048	King et al. (2012b)
Orthopaedic surgery	France and Germany	Single SC injection pre‐op	101	46 (meloxicam)	Global investigator score	Relative efficacy of robenacoxib/meloxicam 1.03 (0.85–1.23) *p* = .76	Speranza et al. (2015)
Soft tissue surgery	Japan	Single SC injection pre‐op	67	29 (meloxicam)	Total clinician score	Relative efficacy of robenacoxib/meloxicam 1.47 (1.19–1.78) *p* = .0003	Kamata et al. (2012)
Ovariohysterectomy	Italy	Single SC injection pre‐op	10 (+10 with buprenorphine)	10 (buprenorphine)	Not defined	Lower pain scores with robenacoxib vs buprenorphine	Staffieri et al. (2013)
Ovariohysterectomy	Thailand	Single oral pre‐op	8	8 (tolfenamic acid) 8 control	Not defined	Lower pain scores with robenacoxib and tolfenamic acid versus control (*p* < .01)	Thengchaisri and Phuwapallanachan (2015)
Ovariohysterectomy	Thailand	Oral pre‐op and 24 and 48 h post‐op	10	10 (tolfenamic acid) 10 (placebo)	Not defined	Lower pain scores with robenacoxib and tolfenamic acid versus placebo (*p* < .05)	Sattasathuchana et al. (2018)

Note

Studies are listed in decreasing order of sample size.

^a^ Maximum number of cats included in analysis, for example for demographic or safety sample. Numbers may have been lower for certain analyses, for example efficacy or per‐protocol sample.

^b^ For King et al. (2016c, 2012b), results are the number (%) of cases rated as treatment success in each group plus the *p* value for comparison of robenacoxib with placebo. For Kamata et al. (2012) and Speranza et al. (2015), which were non‐inferiority studies, results are the relative efficacy of robenacoxib/meloxicam (95% confidence interval) plus the *p* value.

^c^ Treatment success was defined as no need for rescue analgesia.

**TABLE 17:** Summary of clinical trials with robenacoxib in cats with musculoskeletal disorders

Indication	Country	Dosage regimen	Number of cats^a^		Primary endpoint (efficacy)		References
			Robenacoxib	Control	Variable	Results^b^	
Chronic musculoskeletal disorders (safety analysis)	France, UK and USA	Oral for 4–12 weeks	222	227 (placebo)	Incidence of adverse events	Risk of ≥1 adverse event robenacoxib/placebo: 1.15 (0.93–1.43) *p* = .20	King et al. (2021)
Osteoarthritis (safety analysis)	USA	Oral for 4 weeks	95	99 (placebo)	None defined	≥1 adverse event reported in 37 of 95 cats (robenacoxib) versus 33 of 99 cats (placebo) *p* = .46	King et al. (2016a)
Acute musculoskeletal disorders	France and UK	Oral for 5–6 days	56 (once daily) 51 (twice daily)	48 (ketoprofen)	Investigator global assessment score	Relative efficacy of robenacoxib/ketoprofen: 1.004 (0.955–1.056) *p* = .87 (once daily); 1.018 (0.968–1.072) *p* = .48 (twice daily)	Giraudel et al. (2010)
Acute musculoskeletal disorders	Japan	Oral for 5–6 days	47 (once daily)	21 (ketoprofen)	Total clinical score	Relative efficacy of robenacoxib/ketoprofen: 1.151 (0.872–1.494) *p* = .30	Sano et al. (2012)
Degenerative joint disease	USA	Oral for 6 weeks	37	36 (placebo)	Not defined (pilot study)	Robenacoxib was superior (*p* <.05) to placebo in certain analysis (activity and subjective scores)	Adrian et al. (2021)

Note

Studies are listed in decreasing order of sample size.

^a^ For Adrian et al. (2021), numbers are cats enrolled and randomized. Lower numbers were analysed for efficacy in the per‐protocol sample. An additional 36 cats received robenacoxib for 3 weeks followed by placebo for 3 weeks.

^b^ Giraudel et al. (2010) and Sano et al. (2012) were non‐inferiority studies, results are the relative efficacy of robenacoxib/ketoprofen (95% confidence interval) plus the *p* value.

The overall conclusion is that robenacoxib is effective for the selected indications with a treatment effect size comparable to reference NSAIDs.

USA studies evaluated efficacy in soft tissue surgery in dogs and surgery in cats for neutering and front‐limb onychectomy. Robenacoxib or placebo were administered prior to surgery and for two days post‐operatively. Prior to surgery, all animals received butorphanol. Cats additionally had bupivacaine forelimb 4‐point regional nerve blocks. The primary endpoint was a requirement for rescue analgesia. In all studies, the proportion of animals requiring rescue was significantly lower with robenacoxib than with placebo (*p* = .006–.048). The treatment effect size (superiority to placebo) was larger in cats (22.0 and 29.8%) than in dogs (12.4 and 15.6%). The cause of this difference is not known. The findings support earlier conclusions that NSAIDs are efficacious, but that multi‐modal therapy is required for optimal peri‐operative pain and inflammation control (Epstein et al., 2015).

**FIGURE 11:**
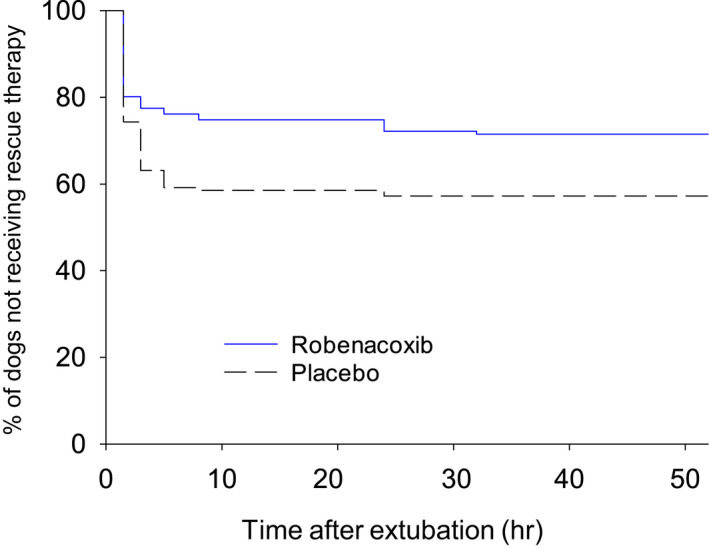
Kaplan–Meier plot of time to rescue analgesia therapy in dogs administered robenacoxib or placebo SC once prior to soft tissue surgery and then for two days post‐surgery. Extubation was at time 0 h. Differences between groups were significant with log‐rank (*p* = .010), generalized Wilcoxon (*p* = .015) and likelihood ratio (*p* = .001) tests (Friton et al., 2017a). Similar results were obtained with tablet PO (Friton et al., 2017b)

**FIGURE 12:**
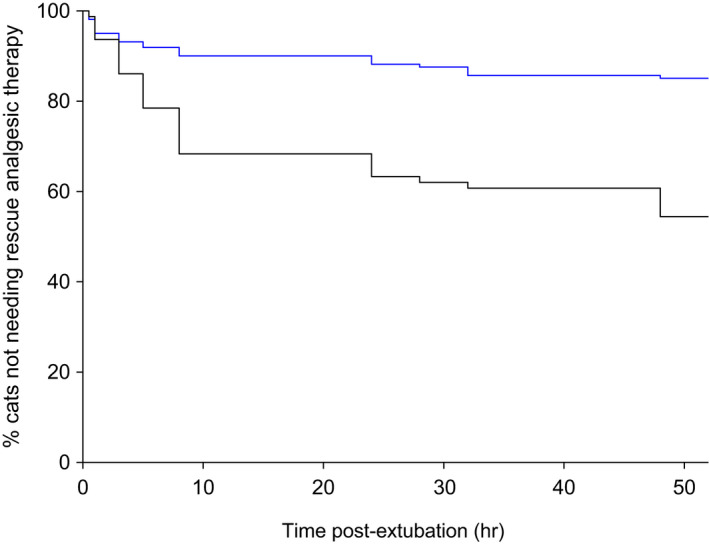
Kaplan‐Meier plot of time to rescue analgesia therapy in cats administered robenacoxib (blue) and placebo (black) PO once prior to orthopaedic and soft tissue surgery and then for two days post‐surgery. Extubation was at time 0 h. The risk of receiving rescue therapy was significantly (*p* < .0001) lower with robenacoxib (King et al., 2012b)

As required by FDA‐CVM, the primary statistical analysis involved comparison of rescue therapy frequency, using a general linear mixed model. Additional time‐to‐event analysis demonstrated that: (1) there is a significant treatment effect (*p* = .046 to < .0001, log‐rank test); and (2) once‐daily dosing provided efficacy over the dosing interval (24 h) (Figures 11, 12).

Company‐sponsored studies in the EU and Japan compared robenacoxib with a positive control: meloxicam for surgery (cats and dogs), carprofen for canine OA and ketoprofen for feline acute musculoskeletal disorders. Non‐inferiority was established in all eight studies with the chosen non‐inferiority margin (δ) of 0.25 (Edamura et al., 2012; Kamata et al., 2012; Sano et al., 2012; Speranza et al., 2015) or 0.20 (Giraudel et al., 2010; Gruet et al., 2011, 2013; Reymond et al., 2012). The δ should represent the maximum difference between the treatments, which is acceptable, and be less than the comparator drug's proven effect size compared with placebo (Freise et al., 2013). In most cases, there were insufficient or no relevant published data to robustly justify the predetermined δ values, which, although commonly used in veterinary trials, were not highly demanding (Freise et al., 2013). In addition, in all studies efficacy was assessed using subjective scoring criteria with possible low discriminating power. Therefore, none of the comparative field studies with robenacoxib had high probability to detect clinically relevant differences from the comparator. The limitations of non‐inferiority studies are well known, but they are the favoured method to support drug registrations in many countries. They avoid the need for a placebo group, which is especially problematic for animal pain studies. Robenacoxib achieved efficacy at least equivalent to reference NSAIDs. Moreover, mean relative efficacy of robenacoxib/control exceeded 1 in 7 of the 8 studies.

For studies involving surgery, robenacoxib had superior efficacy to meloxicam in one cat study (*p* = .0003) (Kamata et al., 2012) and higher mean, but non‐significant, efficacy in three further studies, two in dogs and one in cats (Gruet et al., 2011, 2013; Speranza et al., 2015). Differences between robenacoxib and meloxicam were probably attributable to a faster onset of action of robenacoxib, as previously found in the dog urate synovitis model (Borer et al., 2017; Schmid et al., 2010).

**FIGURE 13:**
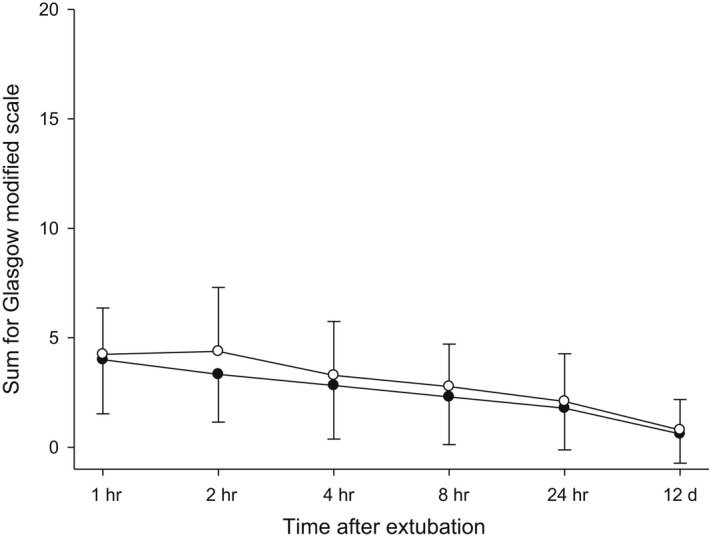
Modified Glasgow pain scale (i.e. without score for mobility) for dogs administered robenacoxib or meloxicam SC once prior to orthopaedic surgery and then PO daily for up to 15 days. Robenacoxib (*n* = 97 [black circles]) or meloxicam (43 [white circles]). Extubation was at time 0 h. The scale ranged from 0 (best) to 20 (worst). Data are mean ± SD (Gruet et al., 2011)

For surgery claims, superiority to placebo in four studies and to meloxicam in one study, together with non‐inferiority to meloxicam in the remaining three studies, clearly demonstrates the efficacy of robenacoxib (Figure 13, Tables 14 and 16). No significant differences in tolerability variables were obtained between groups in all surgery studies. Treatment durations (1–15 days) and group numbers (up to 175 animals per group) suffice to indicate no major differences between the drugs.

Feline acute musculoskeletal disorder studies were small‐scale (21–56 animals per group) and treatment durations short (5–6 days) (Giraudel et al., 2010; Sano et al., 2012). Although non‐inferior efficacy was proven, the studies had insufficient power to differentiate differences in efficacy and safety between robenacoxib and ketoprofen.

**FIGURE 14:**
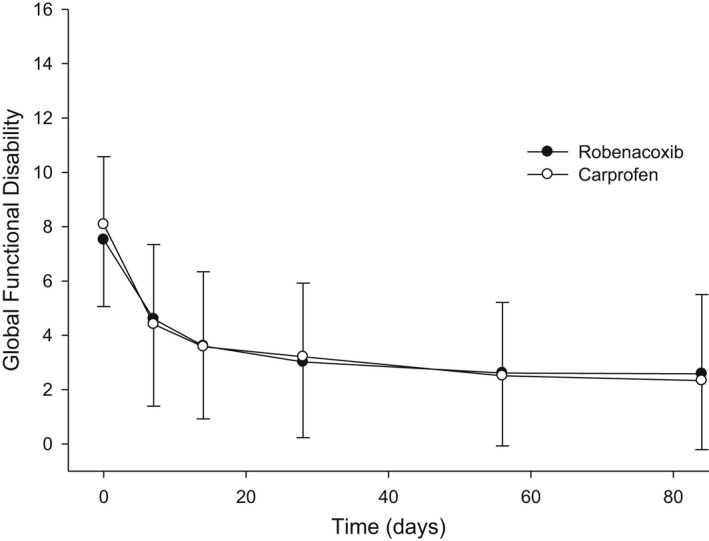
Global functional disability, assessed by the veterinary investigator in dogs with osteoarthritis administered robenacoxib or carprofen PO for up to 3 months. The scale ranged from 0 (best) to 16 (worst). Data are mean ± SD (Reymond et al., 2012)

Whilst canine OA is a major indication for NSAIDs, available data for robenacoxib are limited. A small Japanese study included 32 dogs, 21 receiving robenacoxib and 11 receiving carprofen (Edamura et al., 2012), and they were monitored for only 28 days. An EU study included 186 animals (robenacoxib *n* = 125, carprofen *n* = 63 dogs), and treatment time was 3 months (Reymond et al., 2012). In both studies, there were no significant differences between the drugs for efficacy and safety; both robenacoxib and carprofen provided improvement in veterinarian and owner subjective scores relative to baseline (Figure 14). Neither study incorporated a placebo group; therefore, for both drugs a caregiver placebo effect may have contributed to responses (Conzemius & Evans, 2012).

In the EU study, efficacy was similar for robenacoxib and carprofen (1.00, 95% confidence interval (CI) 0.837–1.19) when robenacoxib was administered without food. There were no differences in adverse event incidence between groups (robenacoxib 46%, carprofen 52%, *p* = 0.44). Hepatic adverse events were reported with both robenacoxib (*n* = 3, 2.4%) and carprofen (*n* = 2, 3.2%).

In conclusion, no sufficiently powered field studies are available to conclude on the relative efficacy and safety in dogs with OA of robenacoxib compared with other NSAIDs. Large studies in humans with OA (respectively 7,111 and 18,325 patients) identified that the COX‐2‐selective drugs etoricoxib and lumiracoxib had significantly better gastrointestinal tolerability than less or non‐selective NSAIDs (Baraf et al., 2007; Schnitzer et al., 2004).

The sponsor company conducted four clinical trials in cats with CMSD, including DJD and OA. To date, efficacy data have been published from one of these studies (Adrian et al., 2021). These preliminary findings indicate some improvement in activity and subjective scores in cats with DJD, but more definitive studies are required.

Safety data from a study in 193 cats with OA indicated no differences between robenacoxib and placebo administered for 28 days (King et al., 2016a). More recently, pooled analysis of safety variables from four clinical trials in cats with CMSD comparing placebo (*n* = 227) with robenacoxib (*n* = 222), administered for 4–12 weeks, indicated that the proportion of cats with at least one reported AE did not differ (*p* = .15) between robenacoxib (106/222, 47.7%) and placebo (93/227, 41.0%) (King et al., 2021). The relative risk was 1.15 (95% CI 0.93–1.43). Together with clinical chemistry and haematology data, the data indicated a good safety profile of robenacoxib in cats with CMSD, with no signal for harm to any organ. Based on sample size, the study had 89% probability to detect AEs with a true incidence ≥1%. The study was underpowered to detect less frequent (<1%) adverse effects. Moreover, application to general practice is limited by the fact that cases with severe and uncontrolled concomitant diseases were excluded.

Seven field studies have been conducted by groups independent of the sponsor company: one open‐label (Bennett et al., 2013) and the remainder comparator‐controlled (Bendinelli et al., 2019; Giorgi et al., 2012; Sattasathuchana et al., 2018; Stabile et al., 2019; Staffieri et al., 2013; Thengchaisri & Phuwapallanachan, 2015). Although these independent studies add to the data set, all were small‐scale (*n* = 5–30 per group).

## 6 DISCUSSION AND CONCLUSIONS

The coxibs were developed with the objective of retaining the efficacy of non‐selective NSAIDs but providing greater safety, particularly for the gastrointestinal tract (Flower, 2003). A large body of evidence from rodent studies and human clinical trials supports the hypothesis that some selective COX‐2 inhibitors cause less gastrointestinal tract ulceration and bleeding than non‐selective agents (Baraf et al., 2007; King et al., 2009; Moore et al., 2006; Schnitzer et al., 2004). For example, there was a 79% reduction in upper gastrointestinal tract complications with lumiracoxib compared to naproxen and ibuprofen in human OA patients (Schnitzer et al., 2004).

However, the coxibs have not met all expectations. Some were withdrawn from human use because of cardiovascular and/or skin (rofecoxib, valdecoxib) or liver (lumiracoxib) toxicity concerns. Moreover, the benefits of some coxibs were either limited or not proven. It is now recognized that COX‐1 may contribute to pain and inflammation (Wallace et al., 2000); COX‐2 is present constitutively in several tissues, exerting physiological roles including tissue healing (Chen & Dragoo, 2013); and both COX isoforms are likely to provide renoprotection in the presence of hypotension and hypovolaemia (Cheng & Harris, 2004).

In veterinary medicine, it may appear to some prescribers that coxibs offer no proven benefit over non‐selective NSAIDs. Furthermore, the cardiovascular side‐effects, which are specific to human beings but which, for physiological reasons, are not applicable in dogs and cats, have tarnished the reputation of coxibs as a class. In consequence, their prescription in human medicine is now limited (Luo et al., 2005). As explained by Katz, coxibs as a group were subjected to reverse bias in some medical journals (Katz, 2013). The current coxib knowledge base in veterinary medicine makes it possible to conclude a more objective, scientifically sound opinion on coxibs in general, and robenacoxib in particular.

No safety advantage of coxibs over older NSAIDs in dogs and cats can be concluded from post‐marketing adverse event reports, for example from the UK (Hunt et al., 2015). Actually, the frequency of emesis, lethargy and death was somewhat higher with coxibs. However, it must be recognized that passive pharmacovigilance studies are subject to many severe biases, which can render their conclusions uncertain. This is particularly the case for the so‐called channelling bias, in which claimed advantages of a new drug may channel it to patients with pre‐existing morbidities, with the consequence that adverse effects can be incorrectly attributed to the newer drug. Channelling towards high‐risk gastrointestinal patients was demonstrated in the incidence of gastrointestinal haemorrhage in human users of meloxicam and coxibs (‘newer NSAIDs’) compared with older, non‐selective, NSAIDs (MacDonald et al., 2003). Adjusting for risk factors reduced the relative risk of gastrointestinal haemorrhage for meloxicam and coxibs versus older non‐specific NSAIDs to 0.84 (95% CI 0.60, 1.17) and 0.36 (0.14, 0.97), respectively. Therefore, after correcting for channelling bias, coxibs (but not meloxicam) were associated with a significantly lower risk of gastrointestinal haemorrhage than older non‐specific NSAIDs.

It is important to recognize that there is no reason why all coxibs should have the same safety profile, as they have markedly differing PD and PK profiles. For example, robenacoxib has a much smaller volume of distribution than firocoxib (see Pharmacokinetics, Section 3) resulting in less exposure of intracellular organelles, whilst distributing selectively to inflamed tissues. Moreover, robenacoxib has a much shorter half‐life (less than 2 h) than mavacoxib (17.3 days), because of a much slower clearance of mavacoxib (2.7 ml/kg/h) (Lees et al., 2015). Cimicoxib and mavacoxib exhibit PK polymorphism in dogs, but the possible clinical impact in terms of efficacy and safety is unknown. Pharmacodynamic profiles, specifically COX‐2 selectivity in target species, also vary considerably between coxibs, from 22:1 for mavacoxib to 384:1 for firocoxib in dogs (IC_50_ COX‐1: IC_50_ COX‐2 ratio), with, to our knowledge, no comparable published data for cimicoxib, enflicoxib and vitacoxib (Lees et al., 2015; McCann et al., 2004). Data on COX‐2 inhibition with sparing of COX‐1 at recommended dosages in the target species have been published for robenacoxib in dogs and cats (see Section 2.1, Pharmacodynamics). Similar data have not been published, to our knowledge, for cimicoxib, enflicoxib, mavacoxib and vitacoxib. The potential clinical relevance of the many PD differences is unknown, and can be resolved only by well‐designed and powered comparative clinical trials. As noted previously, many comparative NSAID veterinary field studies are underpowered (see Section 5, Efficacy and Safety in Clinical Use).

In healthy Beagle dogs, robenacoxib had a high safety margin, at dosages of up to 40 mg/kg for 28 days and up to 10 mg/kg for 6 months. Safety has also been reported in cross‐bred hounds. Comparative studies with diclofenac, which has lower COX‐2 selectivity and a smaller safety margin in rats and dogs, led to the conclusion that the higher COX‐2 selectivity of robenacoxib contributed to improved safety. However, in clinical use with a treatment time only up to 15 days, no safety advantage of robenacoxib compared with meloxicam was demonstrated in dogs undergoing surgery, despite relatively large sample sizes (up to 118 dogs per group). Similarly, in canine OA, field data (maximum 125 dogs per group treated for 3 months) were insufficient to conclude possible differences to carprofen.

In cats, published data are consistent with a superior safety profile of robenacoxib compared with other NSAIDs. In healthy cross‐bred cats, robenacoxib (up to 20 mg/kg for 6 weeks) had a high safety margin with no evidence of toxicity. Although in short treatment duration studies no safety advantage was established under field conditions for robenacoxib compared with meloxicam (for surgery) or ketoprofen (for acute musculoskeletal disorders), it should be noted that only two NSAIDs are registered for long‐term (≥7 days) use in cats, meloxicam and robenacoxib.

For robenacoxib, there were no differences in safety variables compared with negative controls or with placebo in healthy young cats (up to 20 mg/kg for 6 weeks) and cats with CMSD (therapeutic dosage for 4–12 weeks). There are no similar published clinical safety data (from prospective and randomized studies) and target animal safety data, involving long‐term administration of meloxicam in cats. However, the Metacam EU European Public Assessment Report describes gastric and duodenal ulcers at relatively low dosages of meloxicam in healthy cats (0.3 mg/kg on day 1 followed by 0.15 mg/kg for up to 90 days) although dosages of 0.025 to 0.1 mg/kg were well tolerated for 14 days (Anonymous, 2010). It is concluded therefore that robenacoxib has the best proven safety profile of any NSAID in cats. In contrast to dogs, no signal for liver toxicity has been detected in cats; no cases of acute liver toxicity or changes in liver enzymes occurred in laboratory or field studies. This might be explained by the finding that diclofenac toxicity in humans might be due to toxic glucuronidation metabolites (Boelsterli, 2003) and cats have low capacity for glucuronide conjugation of some drugs (van Beusekom et al., 2014).

Whilst it cannot be claimed that the coxibs in general have dramatically transformed pain relief and suppression of inflammation in small animal medicine, they introduced a new group of NSAIDs for clinical use. Within the group, chemical structures and PD and PK profiles vary considerably, thereby adding to the clinician's armamentarium a broad range of novel drugs. This review has focussed on robenacoxib, for which many studies are in the public domain. These have added a rational basis for prescribers’ drug selection for clinical use. Nevertheless, it is proposed that further improved comparative studies be undertaken between NSAIDs of all classes in order to improve their safe and efficacious use.

## CONFLICT OF INTEREST

P. Lees is Emeritus Professor of Veterinary Pharmacology, Royal Veterinary College, University of London. He has acted as Consultant to Novartis Animal Health and supervised the PhD studies of J.M. Giraudel and L. Pelligand, which focussed on robenacoxib. P.‐L. Toutain is formerly Professor of Veterinary Pharmacology, University of Toulouse, and is currently Distinguished Visiting Professor at the Royal Veterinary College, University of London. He co‐supervised the PhD studies of J.M. Giraudel. J. Elliott is Professor of Veterinary Clinical Pharmacology, Royal Veterinary College, University of London. He acted as a consultant for Elanco Animal Health (Advisory Boards), received grant funding from Elanco from their Fellowship scheme and co‐supervised the PhD studies of L. Pelligand. J.M. Giraudel's PhD thesis focussed on the pre‐clinical pharmacology of robenacoxib in the cat. L. Pelligand's PhD thesis focussed on the comparative pharmacology of robenacoxib. J.M. Giraudel and J.N. King were formerly employed by Novartis Animal Health and Elanco Animal Health, which distributes and markets robenacoxib (Onsior^®^).

## ANIMAL WELFARE AND ETHICS STATEMENT

Since this is a review article, no new data are presented. Animal welfare and ethics statements are contained, where appropriate, in the original references. [Correction added on 6 September 2022, after first online publication: The Animal Welfare and Ethics Statement was included in this current version.]

## AUTHOR CONTRIBUTIONS

P. Lees and J.N. King drafted the first version of this review, and revised several further drafts and prepared the final version with P‐L. Toutain. All other authors contributed to several drafts and the final version of the article.

## Data Availability

As this is a review article, no new data are presented.
